# Strong internal resonance in a nonlinear, asymmetric microbeam resonator

**DOI:** 10.1038/s41378-020-00230-1

**Published:** 2021-01-26

**Authors:** Keivan Asadi, Junghoon Yeom, Hanna Cho

**Affiliations:** 1 Department of Mechanical and Aerospace Engineering, The Ohio State University, Columbus, OH 43210 USA; 2 Department of Mechanical Engineering, Michigan State University, East Lansing, MI 48824 USA

**Keywords:** NEMS, Electrical and electronic engineering

## Abstract

Exploiting nonlinear characteristics in micro/nanosystems has been a subject of increasing interest in the last decade. Among others, vigorous intermodal coupling through internal resonance (IR) has drawn much attention because it can suggest new strategies to steer energy within a micro/nanomechanical resonator. However, a challenge in utilizing IR in practical applications is imposing the required frequency commensurability between vibrational modes of a nonlinear micro/nanoresonator. Here, we experimentally and analytically investigate the 1:2 and 2:1 IR in a clamped–clamped beam resonator to provide insights into the detailed mechanism of IR. It is demonstrated that the intermodal coupling between the second and third flexural modes in an asymmetric structure (e.g., nonprismatic beam) provides an optimal condition to easily implement a strong IR with high energy transfer to the internally resonated mode. In this case, the quadratic coupling between these flexural modes, originating from the stretching effect, is the dominant nonlinear mechanism over other types of geometric nonlinearity. The design strategies proposed in this paper can be integrated into a typical micro/nanoelectromechanical system (M/NEMS) via a simple modification of the geometric parameters of resonators, and thus, we expect this study to stimulate further research and boost paradigm-shifting applications exploring the various benefits of IR in micro/nanosystems.

## NEMS Resonators: Achieving strong internal resonance

Achieving strong ‘internal resonance’ within a simple nanoelectromechanical resonator opens doors to further research and practical applications. Hanna Cho, and a team from The Ohio State University and Michigan State University, United States, describe in their new paper how internal resonance (IR) offers a “unique pathway” to steer vibrational energy, with applications in signal detection and simultaneous physical property sensing. Robust achievement of IR has proved challenging, with the property first realized in the early 2010s. The team now describe the robust generation of IR by a “relatively simple” nanoelectromechanical resonator, and the design parameters that can be manipulated to alter resonance behaviors. The team hope that their blueprints can act as a stepping stone for deeper investigation into the exploitation of IR for various applications.

## Introduction

The performance of resonator-based micro/nanoelectromechanical systems (M/NEMSs) strongly relies on the dynamic characteristics of their mechanical element, the micro/nanoresonator^1^. The advancement of micro/nanotechnology indispensably leads to size reduction and *Q* factor enhancement in resonators, making their dynamic responses transit easily from the linear to nonlinear regime^2,3^. Even though it is more difficult to predict and design nonlinear behaviors in micro/nanosystems, nonlinearity can introduce intriguing features that are not attainable in a linear setting. These features include hysteresis phenomena, multivalued responses, amplitude bifurcations, amplitude-frequency dependencies, and various types of nonlinear resonances^4–7^. Along with the evolution in micro/nanotechnology, researchers have devoted vigorous efforts to exploring these nonlinear characteristics in various applications, such as mass sensing^8,9^, bio/chemical detection^10,11^, inertial sensors^12–15^, radio frequency communication circuits^16–18^, logic gates^19–22^, and optical resonators^23–26^.

Among the various nonlinear characteristics, one remarkable category belongs to intermodal coupling and nonlinear energy transfer, where two or more vibrational modes interact with each other^5,27^. The intermodal coupling strength can be significantly enhanced when the following conditions for the so-called internal resonance (IR) are satisfied in a nonlinear system: (i) the resonant frequencies of distinct vibrational modes are commensurate or nearly commensurate with each other; (ii) a proper type of nonlinearity in accordance with frequency commensurability exists^5^. When the condition of frequency commensurability is satisfied in a nonlinear system, the superharmonic or subharmonic term of an externally resonated mode coincides with another mode(s) of the system and thus can internally resonate with the associated undriven mode(s). When the IR is triggered, strong internal coupling results in an effective and fast energy flow between internal modes, and the resonance behaviors and characteristics are drastically altered. Thus, the IR provides a unique pathway to steering vibrational energy within a single unit mechanical system, serving as the basis for various applications and fundamental studies. For instance, the transfer of excessive energy through strong intermodal coupling can stabilize the frequency fluctuations in a micro/nanomechanical oscillator^28–31^ and enable the detection of angular rate signals in Coriolis vibratory gyroscopes^32,33^. The IR mode can also be employed as an additional sensing channel to measure two different physical quantities simultaneously^34–36^. In addition, the faster energy exchange between the internal modes than that caused by the environmental source can provide an efficient route to engineering the intrinsic dissipation of an oscillator^37–39^.

The remaining challenge in taking advantage of the benefits of IR in practical applications is to robustly generate and tailor strong IR in the resulting dynamics. Indeed, the experimental realization of IR in micro/nanoresonators was not achieved until the early 2010s, while theoretical investigations have been extensively reported in the literature^40–48^. This delay emanates from the fact that resonator designs of simple uniform geometries (e.g., prismatic beams) do not typically satisfy the commensurability condition. Most of the previous resonators intentionally designed to implement IR were based on atypical structural shapes and modes for M/NEMSs. For example, T-shaped beams^49,50^ and H-shaped plates^41,51^ were considered to enforce 1:2 IR; the flexural and extensional modes were internally coupled in a two-beam system^30^; and the flexural and torsional modes were internally coupled in a clamped-clamped beam system^28,39^. The success of these examples comes at the price of unconventional structures and modes imposing additional complications in the design of actuation and transduction electrodes. Thus, this paper aims to provide a strategy that can integrate a strong IR in a relatively simple nonprismatic beam resonator by coupling two flexural modes. We provide a detailed experimental and theoretical analysis of 1:2 and 2:1 IR systems and discuss effective design parameters that qualitatively alter resonance behaviors.

## Results

### Experimental characterization of internal resonance

**Fig. 1:**
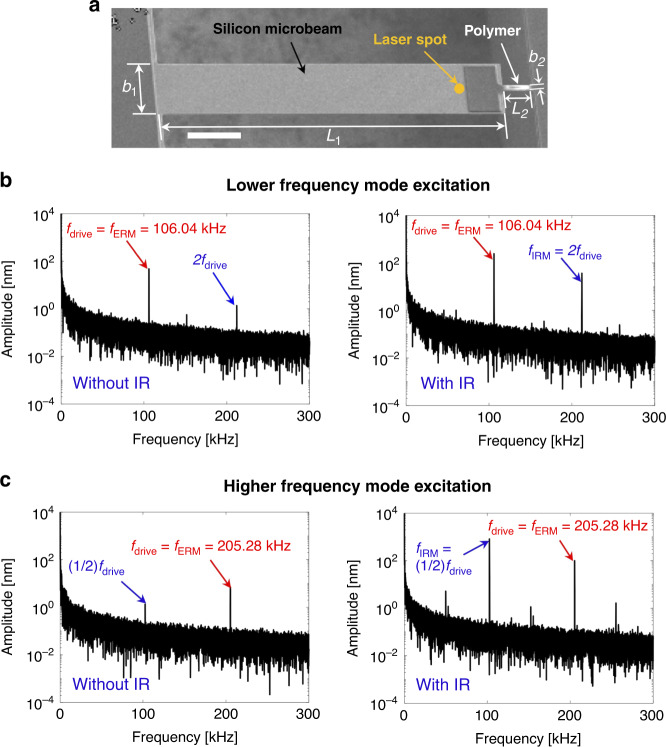
SEM image of the resonator and its FFT responses. **a** Scanning electron micrograph of a nonlinear microresonator whose 2nd and 3rd flexural modes are close to a 1:2 ratio ($f_1 \cong 42\,{\mathrm{kHz}},\,f_2 \cong 107\,{\mathrm{kHz}}$, and $f_3 \cong 214\,{\mathrm{kHz}}$). The scale bar is 100 μm. **b**, **c** Comparison of the harmonic content in FFT-based spectral responses between the cases without (left column) and with (right column) IR, which shows that IR acts as a mechanism that amplifies the undriven IRM by tunneling the energy from the ERM. For the LME with $f_{{\mathrm{drive}}} = 106.04\,{\mathrm{kHz}}$ in (**b**), the IRM amplitude is increased from 1.34 to 36.35 nm as the IR is triggered, while the ERM amplitude increases from 30.17 to 242.5 nm. For the HME with $f_{{\mathrm{drive}}} = 205.28\,{\mathrm{kHz}}$ in (**c**), the IRM amplitude is increased from 1.35 to 825.8 nm, while the ERM amplitude increases from 7.1 to 99.78 nm. Note that the undriven IRM has a higher oscillation amplitude than that of the directly driven ERM for HME

A scanning electron microscopy (SEM) image of the microresonator designed to implement IR is shown in Fig. 1a. The system consists of a silicon microbeam spanned to a firm substrate by a small polymer component. In this design, the axial stiffness of the attached polymer component is ~40 times lower than that of the Si microbeam. Hence, when the system oscillates, the freestanding polymer component is axially stretched, resulting in geometric nonlinearity^52^. The dimensions of the structural components were deliberately chosen to produce the desired 1:2 ratio between the second and third mode frequencies: the length (*L*), width (*b*), and thickness (*h*) of the silicon microbeam (subscript 1) and polymer coupling (subscript 2) are $L_1 = 500\,\upmu {\mathrm{m}},\,b_1 = 100\,\upmu {\mathrm{m}},\,h_1 = 2\,\upmu {\mathrm{m}}$ and $L_2 = 40\,\upmu {\mathrm{m}},\,b_2 = 12\,\upmu {\mathrm{m}},\,h_2 = 3\,\upmu {\mathrm{m}}$, respectively. The thermomechanical response measured by a laser Doppler vibrometer (LDV) showed that the first three linearized mode frequencies were $f_1 \cong 42\,{\mathrm{kHz}},\,f_2 \cong 107\,{\mathrm{kHz}}$, and $f_3 \cong 214\,{\mathrm{kHz}}$ and that the second and third mode frequency values satisfied the 1:2 relation of commensurability. The strong geometric nonlinearity in the heterogeneous nonprismatic design^52^, combined with the 1:2 ratio between the mode frequencies, triggers the IR in the dynamic response. This outcome implies that the second and third modal responses can be internally coupled if the system is driven hard enough into the nonlinear regime. Thus, in this work, the responses in the second and third modes were monitored when one of these modes was externally driven by applying a single-frequency excitation around one of these two mode frequencies. For the sake of simplicity and clarity, the abbreviations LM and HM are used to denote the lower-frequency (i.e., second) mode and higher-frequency (i.e., third) mode, and ERM and IRM are used to denote the externally resonated (i.e., directly driven) mode and internally resonated mode. We also use LME (LM excitation) and HME (HM excitation) to denote which mode is externally excited.

**Fig. 2:**
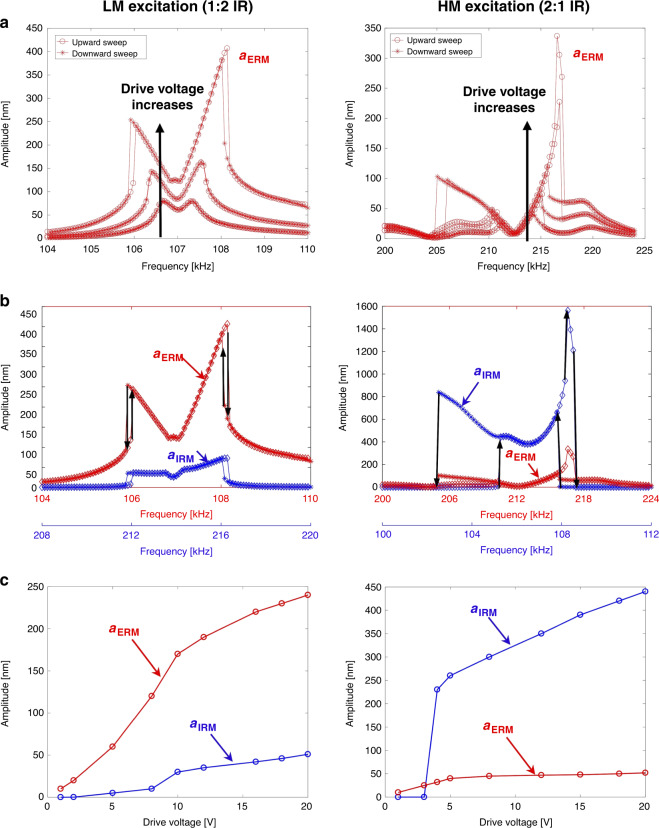
Experimental characterization of 1:2 and 2:1 IR when the LM (in the left column) or HM (in the right column) is driven. **a** ERM amplitudes as a function of the driving frequency show the signature M-shaped IR curves at three different excitation amplitudes. The amplitudes during the upward and downward frequency sweeps are shown as circles and asterisks, respectively. As the excitation amplitude increases, the IR activation range expands, and the hysteresis manifests. **b** Amplitudes of the ERM (at *f*_drive_) and IRM ($f_{{\mathrm{IRM}}} = 2f_{{\mathrm{drive}}}$ or ${\mathrm{f}}_{{\mathrm{IRM}}} = 1/2\,{\mathrm{f}}_{{\mathrm{drive}}}$) showing the coexistence of the two modes in the system when IR is activated. The IR activation range is different depending on the sweeping direction, which results in the hysteresis and jump phenomena marked by the black arrows. **c** Steady-state amplitudes of the ERM and IRM as a function of the driving voltages, which show that there is a threshold energy for the onset of IR. It is clearly shown that the external energy pumped to the ERM is transferred to the IRM once the IR is activated. The energy transfer from the ERM to the IRM leads to the amplitude saturation phenomenon in 2:1 IR

While the existence of subharmonics and/or superharmonics in a nonlinear dynamic response is not an uncommon phenomenon, the IR substantially amplifies those harmonics due to a strong intermodal energy transfer between the engaged modes. From the fast Fourier transformed (FFT) responses shown in Fig. 1b, c, the amplitudes of these harmonics are compared between the cases in which the IR is triggered (right column) and is not triggered (left column). Because the IR is activated only if the input energy is higher than a threshold value (see Fig. 2c), the drive voltage amplitude was tuned to either enter or escape the range of the IR. For LME, at the excitation frequency $f_{{\mathrm{drive}}} = 106.04\,{\mathrm{kHz}}$, the amplitude at the second harmonic was increased from 1.34 to 36.35 nm (see Fig. 1b) when the IR was triggered, while the ERM amplitude was increased from 30.17 to 242.50 nm under the two different drive amplitudes. The amplification in the IRM is more noticeable in the HME at the drive frequency $f_{{\mathrm{drive}}} = 205.28\,{\mathrm{kHz}}$. The comparison of the amplitudes at the subharmonic of order 1/2 in Fig. 1c shows that activation of the 2:1 IR amplified the subharmonic term from 1.35 to 825.8 nm. The IRM amplitude in this 2:1 IR response was even higher than that of the directly driven ERM amplitude, which corroborates the vigorous energy transfer between the internally coupled modes. Note that the smaller peaks in the frequency spectrum in Fig. 1b, c are around the subharmonics and ultraharmonics generated by various types of small nonlinear effects.

Figure 2 shows an experimental characterization of the nonlinear IR frequency responses when the LM (left column) or HM (right column) was externally driven. In Fig. 2a, the frequency responses of the ERM are depicted during the upward (in circles) and downward (in asterisks) frequency sweeps at three different levels of excitation. The results yielded typical M-shaped 1:2 IR response curves. The higher energy input to the system drove the system further into the nonlinear regime and expanded the IR activation range. Eventually, hysteresis phenomena manifested because multiple stable branches coexisted. Figure 2b shows the amplitude of the ERM and IRM with respect to the driving frequency at the highest excitation voltage. As the driving frequency approached the mode frequency from a lower frequency, the ERM amplitude gradually increased. When the energy level of the ERM surpassed a critical value, the IR mechanism was activated in the system, and both the ERM and IRM were amplified. This intermodal nonlinear interaction resulted in vigorous energy exchange between the engaged modes until the drop-jump phenomenon occurred. These IR activation ranges were different depending on the sweeping direction, which demonstrated hysteresis in both the 1:2 and 2:1 IR responses. The hysteresis range was wider in the 2:1 IR, and one extra transition to an upper branch was found before the drop-down transition. Figure 2c shows the ERM and IRM amplitudes with respect to the excitation level at a fixed driving frequency (*f*_drive_ = 107.5 kHz in the LME and *f*_drive_ = 214.2 kHz in the HME). When the LM was driven at voltages lower than 10 V, the ERM amplitude increased linearly with the forcing level, and the IRM amplitude was nearly zero. Due to the intrinsic geometric nonlinearity of the system, a higher (2nd) harmonic existed even when the IR was not triggered. Once IR was activated at a driving voltage of ~10 V, a sudden jump in the IRM amplitude occurred while energy continuously transferred from the directly driven ERM to the undriven IRM. On the other hand, when the HM was externally driven, the so-called amplitude saturation phenomenon was observed beyond a threshold forcing level of ~5 V, where the extra energy applied to the ERM was channeled directly into the undriven IRM and the ERM amplitude remained constant. Comparing the 1:2 IR and 2:1 IR responses, we conclude that the intermodal energy transfer from the ERM to the IRM is more vigorous and effective for the 2:1 IR case, as the amplitude of the IRM exceeds that of the ERM by an order of magnitude.

### Analytical modeling

We developed an analytical model based on the energy method to further understand the underlying dynamics in the nonlinear 1:2 and 2:1 IR systems. The analytical results provide more detailed knowledge of the complex IR dynamics and the modal energy transfer. The patterns of the nonlinear resonances in IR systems drastically change depending on the type of nonlinear coupling (i.e., quadratic or cubic), coupling strength, internal frequency mismatch from the exact commensurability condition, and forcing level. Thus, studying the effective parameters responsible for the unique resonance behaviors is essential to exploit IR in practical systems with the desired resonance features.

To obtain the analytical model, we first defined the transverse displacement of a beam in which both the LM and HM are excited by a base excitation (see Eq. (1) in the “Materials and methods” section). When the base excitation frequency (Ω) is close to the LM frequency (i.e., ${\mathrm{{\Omega}}} = \omega _1 + \eta \sigma _2$, where *η* is a small-scale parameter and σ_2_ is an external frequency detuning parameter), the LM is harmonically driven at an excitation frequency of Ω, and the HM is internally resonated at a frequency of 2Ω. Similarly, for the case of HME, the HM is externally excited at Ω, while the LM is internally resonated at Ω/2. We also imposed an internal frequency mismatch from the exact 1:2 ratio between the LM and HM frequencies to account for any potential deviation from the intended design in the wake of fabrication errors and parameter randomness. In this regard, the relationship between the mode frequencies is expressed with the equation $\omega _2 = 2\omega _1 + \eta \sigma _1$, where *σ*_1_ is an internal frequency detuning parameter. Using the transverse displacement of a beam based upon these settings, the averaged Lagrangian (see Eq. 6) and Lagrange’s equation were obtained to eventually deduce a set of leading-order nonlinear equations governing the modal amplitudes (see Eq. 7). The leading-order governing equations show that each LM or HM itself is modeled as a linear harmonic oscillator with quadratic nonlinear coupling originating from the axial strain (*∈*_*xx*_). The axial stretching brings about the cubic coupling terms between the modal amplitudes of *A*_1_ and *A*_2_ (e.g., $A_1^3,A_2^3,A_1^2A_2,\,A_1A_2^2$) in the strain energy, but only the term of $A_1^2A_2$ remains as the only effective nonlinear term in the time-averaged Lagrangian equation. Solving these equations under the steady-state condition, the resulting dynamic behaviors are analytically characterized under various sets of system parameters to suggest strategies to tailor the complex IR dynamics. The detailed analytical process is outlined in the “Materials and methods” section and in the Supplementary Information.

### IR design parameter

**Fig. 3:**
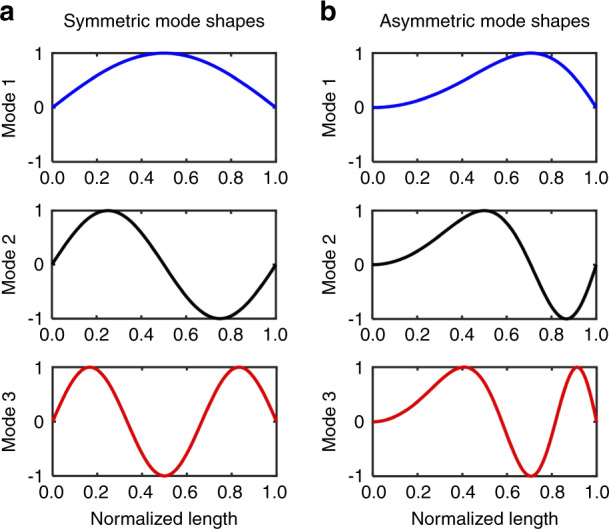
First three flexural mode shapes. **a** A symmetrical configuration $w_n\left(x\right)=\sin\left({\frac{{n\pi}}{L}x}\right)$ and **b** an asymmetrical configuration with $w_n\left(x\right)=\sin\left({\frac{{n\pi}}{L}x^2}\right)$ for $n=1,\,2,\,3$

The nonlinear coupling terms, obtained analytically in Eq. (8) and Eq. (S6), are generated by the pure geometric (stretching) effect and determined by the geometric parameters and linear mode shapes of the engaged modes. Therefore, one can design 1:2 IR systems with the targeted resonance behaviors by tailoring the geometric parameters in Eq. (8). To suggest the design parameters that can effectively incorporate IR into micro/nanomechanical resonators, the effect of the mode shapes is investigated by considering two sets of symmetric and asymmetric mode shapes, expressed by families of the trial functions $w_{n}\left(x\right)=\sin\left(\frac{{n\pi}}{L}x\right)$ and $w_n\left(x\right)=\sin \left({\frac{{n\pi}}{L}x^2}\right)$, respectively, for *n* = 1, 2, 3, as depicted in Fig. 3. Note that these functions, satisfying the zero displacement boundary conditions, are just two simple examples of symmetric and asymmetric mode shapes that are not specifically applied to the resonator in this paper. We perform a similar analysis on more examples of modes, including higher modes and modes of a doubly clamped beam, in Section 2 of the Supplementary Information. The coupling coefficients for these mode shapes calculated using Eq. (8) are shown in Table 1, with the other system parameters set to the same values. The results summarized in Table 1 suggest two notable facts. First, the asymmetric mode shapes provide a stronger intermodal coupling between any of the three modes than that offered by the symmetric mode shapes. Second, the strongest coupling occurs between the second and third modes among the lowest three flexural modes that are relatively readily achievable in practice. These two attributes confirm the validity of the mechanical resonator design in the experimental study, where a 1:2 ratio was implemented between the second and third modes in a heterogeneous nonprismatic beam. Altering the design of a beam resonator from a prismatic (symmetric) to nonprismatic (asymmetric) shape not only offers more freedom to tune the frequency ratio into the required integer but also renders stronger coupling between modes. Even though the current system is rather atypical due to the polymer component, a homogeneous beam (e.g., silicon beam) can also be designed to obtain asymmetric second and third mode shapes with a 1:2 frequency ratio simply by varying the dimension along the beam (e.g., a stepped beam or a tapered beam).

**Table 1:** Nonlinear coefficients in 1:2 IR systems with symmetrical and asymmetrical flexural modes.

	Beam with symmetrical mode shapes			Beam with asymmetrical mode shapes		
	Flexural mode numbers			Flexural mode numbers		
	1st–2nd	1st–3rd	2nd–3rd	1st–2nd	1st–3rd	2nd–3rd
$\left| {\alpha _1} \right|$	0	2.23	3.19	5.65	10.95	18.84
$\left| {\alpha _2} \right|$	0	0.56	0.80	1.29	2.42	4.54

Geometric parameters other than the mode shapes are set to constant values as follows: $\rho = 1,\,\left( {\frac{{\upsilon \mu }}{{1 - 2\upsilon }} + \mu } \right) = 1,\,L = 1,\,b = 0.1,\,{\mathrm{and}}\,h = 0.01$ (see Eq. (8)).

### Analytical results

**Fig. 4:**
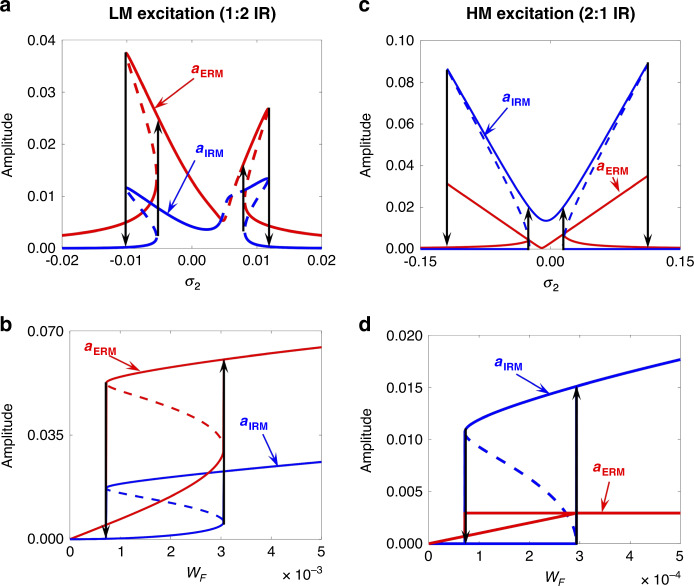
Analytical results of the 1:2 and 2:1 IR when the LM (in the left column) or HM (in the right column) is driven. **a**, **b** Analytical results of the 1:2 IR response with $\omega _1 = 1,\,\omega _2 = 2,\,\zeta _1 = \zeta _2 = 0.001,\,\left| {\alpha _1} \right| = 0.87,\left| {\alpha _2} \right| = 0.25,\,\lambda = 0.1$, and $\sigma _1 = 0.01$. The amplitudes of the ERM (red) and IRM (blue) as a function of the external frequency detuning parameter are shown in (**a**) when $w_F = 5 \times 10^{ - 4}$. The solid and dashed lines represent the stable and unstable branches, respectively, and the arrows show the direction of the jump phenomena. The ERM and IRM amplitudes with respect to the drive amplitude are shown in (**b**) at a fixed driving frequency $\sigma _2 = - 0.015$. **c**, **d** Analytical results of the 2:1 IR response with $\omega _1 = 1,\,\omega _2 = 2,\,\zeta _1 = \zeta _2 = 0.001,\,\left| {\overline {\alpha _1} } \right| = 1.74,\,\left| {\overline {\alpha _2} } \right| = 0.5,\,\bar \lambda = 0.2$, and $\sigma _1 = 0.01$. The ERM and IRM amplitudes as a function of the external frequency detuning parameter are shown in (**c**) when $w_F = 5 \times 10^{ - 4}$. The ERM and IRM amplitudes with respect to the driving amplitude are shown in (**d**) at a fixed driving frequency $\sigma _2 = - 0.02$

The analytical nonlinear amplitudes of the ERM and IRM in 1:2 IR are shown in Fig. 4a as a function of the external frequency detuning parameter, along with their stability (solid lines for stable solutions and dashed lines for unstable solutions). When the IR is not activated in the system, the resonance plot consists of single solution branches. Within the IR activation range $\left( { - 0.01 \,<\, \sigma _2 \,<\, 0.012} \right)$, there are two intervals with two stable and one unstable solution ($- 0.01 \,<\, \sigma _2\, <\, - 0.005$ and $0.007 \,<\, \sigma _2 \,<\, 0.012$) and one interval with a single solution branch $\left( { - 0.005 \,<\, \sigma _2 \,<\, 0.007} \right)$. Near the valley of the M-shaped curve, there is also an interval with one unstable solution in which a continuous energy exchange between the two modes leads to quasi-periodic time responses (see Fig. S3 in the Supplemental Information). The multiple stable solutions result in the signature hysteresis phenomenon. Note that the nonzero internal detuning parameter $\left( {\sigma _1 = 0.01} \right)$ in Fig. 4a makes the ERM and IRM line shapes tilt into asymmetric M-shaped curves (see Fig. S6 for results corresponding to various internal detuning parameters). Figure 4b shows that the activation of IR depends on the energy level applied to the system. When increasing the driving amplitude from zero, there is a nearly linear increase in the ERM and IRM amplitudes until the driving amplitude reaches a critical value of 3.1 × 10^−3^. Beyond this value, the IR is activated and leads to a sudden jump in the amplitudes of both modes. Following that, the ERM and IRM amplitudes steadily grow as a consequence of the continuous intermodal energy exchange. There also exists a hysteresis depending on the force direction due to the multiple values of the stable branches.

Figure 4c shows the analytical nonlinear amplitude responses of the ERM and IRM in 2:1 IR with respect to the external frequency detuning parameter (*σ*_2_) when $\sigma _1 = 0.01$ (see Fig. S6 for results corresponding to various internal detuning parameters). When the IRM has no real solution, the ERM follows the solution of the corresponding linear problem. When the IRM is triggered and has two real solutions, the ERM follows the solution of the first equation in Eq. (14). Figure 4d presents the saturation phenomenon in 2:1 IR, where the ERM amplitude does not depend on the forcing level. Increasing the driving amplitude from zero in Fig. 4d makes the ERM amplitude grow linearly, whereas the IRM amplitude remains zero. When the driving amplitude reaches 3 × 10^−4^, the IRM amplitude sharply rises to a stable branch due to the activation of IR in the dynamic response. A further increase in the driving energy does not affect the ERM amplitude (see the 1st equation of Eq. S8, which does not include a forcing term in *a*_2_) but is purely used to increase the IRM amplitude. The comparison of the responses in the 1:2 and 2:1 IR scenarios again confirms that the internal energy transfer is more vigorous in the 2:1 IR. The analytical results shown in Fig. 4 are in good agreement with the experimentally obtained results shown in Fig. 2. Note that the blunt jump shown in Fig. 2c, compared to the case in Fig. 4b, is speculated to be the result of insufficient experimental data points around the threshold voltage.

**Fig. 5:**
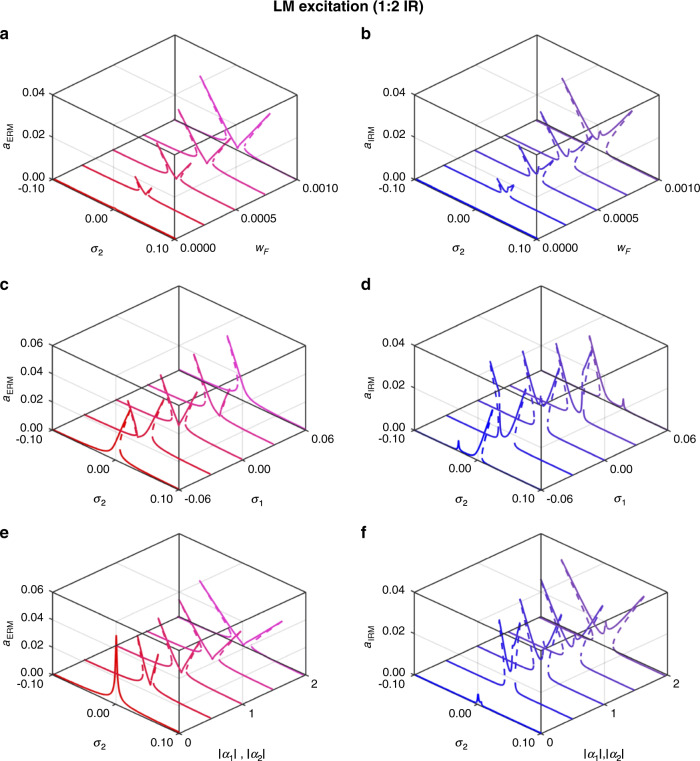
Parametric study of the 1:2 IR The left column shows the ERM amplitude, while the right column shows the IRM amplitude. The effect of the excitation level is shown in (**a**) for $\omega _1 = 1,\,\omega _2 = 2,\,\zeta _1 = \zeta _2 = 0.001,\,\left| {\alpha _1} \right| = 0.87,\,\left| {\alpha _2} \right| = 1.75,\,\lambda = 0.1,\,{\mathrm{and}}\,\sigma _1 = 0.01$. When the driving force increases, the IR activation range expands, and the oscillation amplitudes increase. A change in the internal frequency mismatch shown in (**b**) for $\omega _1 = 1,\,\omega _2 = 2,\,\zeta _1 = \zeta _2 = 0.001,\,\left| {\alpha _1} \right| = 0.87,\,\left| {\alpha _2} \right| = 1.75,\,\lambda = 0.1,\,{\mathrm{and}}\,w_F = 5 \times 10^{ - 4}$, alters the overall resonance line shape from a symmetric M-shape to asymmetric ones. The effect of nonlinear coupling constants is shown in (**c**) for $\omega _1 = 1,\,\omega _2 = 2,\,\zeta _1 = \zeta _2 = 0.001,\lambda = 0.1,\,\sigma _1 = 0.01,\,{\mathrm{and}}\,w_F = 5 \times 10^{ - 4}$. A larger nonlinear coefficient enhances the energy transfer between the ERM and IRM as the ERM amplitude decreases and the IRM amplitude increases. The same sets of data plotted in the 2-D graphs can be found in the Supplementary Information

**Fig. 6:**
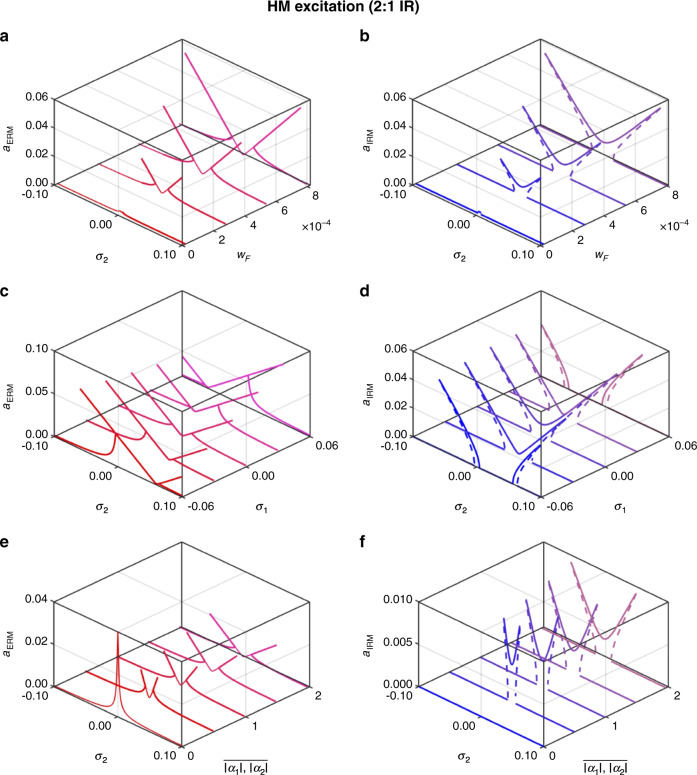
Parametric study of the 2:1 IR. The left column shows the ERM amplitudes, while the right column shows the IRM amplitudes. The effect of the excitation level is shown in (**a**) for $\omega _1 = 1,\,\omega _2 = 2,\,\zeta _1 = \zeta _2 = 0.001,\,\left| {\overline {\alpha _1} } \right| = 0.85,\,\left| {\overline {\alpha _2} } \right| = 1.75,\,\bar \lambda = 0.2,\,{\mathrm{and}}\,\sigma _1 = 0$. When the driving force increases, the IR activation range expands, and the oscillation amplitudes increase. A change in the internal frequency mismatch, shown in (**b**) for$\omega _1 = 1,\,\omega _2 = 2,\,\zeta _1 = \zeta _2 = 0.001,\left| {\overline {\alpha _1} } \right| = 0.85,\,\left| {\overline {\alpha _2} } \right| = 1.75,\,\bar \lambda = 0.2,{\mathrm{and}}\,w_F = 7e - 4$, alters the overall resonance line shape from a symmetric M-shape to asymmetric ones. The effect of nonlinear coupling constants is shown in (**c**) for $\omega _1 = 1,\,\omega _2 = 2,\,\zeta _1 = \zeta _2 = 0.001,\,\bar \lambda = 0.2,\sigma _1 = 0,\,{\mathrm{and}}\,w_F = 2e - 4$. A larger nonlinear coefficient enhances the energy transfer between the ERM and IRM as the ERM amplitude decreases and the IRM amplitude increases. The same sets of data plotted in the 2-D graphs can be found in the Supplementary Information

It is of significance to study how different parameters can qualitatively change the resonance behavior in IR systems. We conducted a parametric study to examine the effect of the driving amplitude (*w*_*F*_), internal frequency detuning (*σ*_1_), and nonlinear coefficients (*α*_*i*_) on the IR when they are varied over the ranges $0 \le w_F \le 0.001,\, - 0.06 \le \sigma _1 \le 0.06,\,{\mathrm{and}}\,0 \le \left| {\alpha _1} \right|,\left| {\overline {\alpha _1} } \right| \le 2$. Figures 5 and 6 show the results for the 1:2 and 2:1 IR cases, respectively, in which the ERM amplitudes are shown in the left column and the corresponding IRM amplitudes are shown in the right column (see Figs. S4–S6 for closer views of the ERM and IRM amplitudes in the 2-D amplitude-frequency planes). The variation in the driving amplitude *w*_*F*_ in both the 1:2 and 2:1 IR cases indicates that a larger forcing level elevates the oscillation amplitude and expands the IR bandwidth (see Figs. 5a and 6a). The variation in the internal frequency mismatch *σ*_1_ alters the overall line shape of the amplitude responses, as shown in Figs. 5b and 6b. For both 1:2 and 2:1 IR, when there is no internal frequency mismatch (*σ*_1_ = 0), the response exhibits the signature M-shaped resonance curve, which is tilted into an asymmetric curve for ${\sigma_1 \neq 0}$. For the 1:2 IR, the large negative (*σ*_1_ = −0.06) and positive (*σ*_1_ = 0.06) values for the internal frequency mismatch generate hardening- and softening-type resonance line shapes, respectively, in both the ERM and IRM. The tilting direction depends on the sign of the internal frequency mistuning and is reversed in the 2:1 IR. Finally, the influence of the nonlinear coupling coefficients *α*_*i*_ is examined in Figs. 5c and 6c. Larger coupling coefficients in the system enhance the intermodal coupling between the IRM and ERM, mainly affecting the bandwidth of the IR activation range rather than the IRM amplitude (see Fig. S5).

**Fig. 7:**
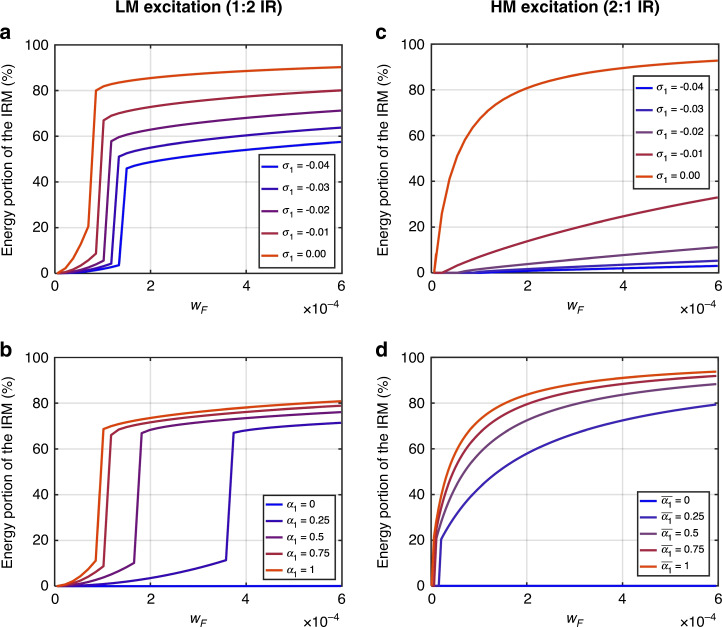
Energy portion of the IRM. The ratio of energy transferred to the IRM to the total energy in both the ERM and IRM is plotted as a function of *w*_*F*_ when the parameters of *σ*_1_ and *α*_*i*_ are varied. The left column (**a**, **b**) shows the cases for 1:2 IR, and the right column shows the cases for 2:1 IR. Other parameters used in this analysis are$\omega _1 = 1,\,\omega _2 = 2,\,\zeta _1 = \zeta _2 = 0.001,\,\left| {\alpha _1} \right| = 0.87,\,\left| {\alpha _2} \right| = 1.75,\,\lambda = 0.1,\left| {\overline {\alpha _1} } \right| = 0.87,\,\left| {\overline {\alpha _2} } \right| = 1.75,\,\overline \lambda = 0.2,\,{\mathrm{and}}\,\sigma _2 = 0.005$

We also investigate the effects of the internal detuning parameter *σ*_1_ and nonlinear coefficients *α*_*i*_ on how much energy is transferred to the IRM. Figure 7 plots the ratio of the IRM energy to the total system energy as a function of the driving amplitude *w*_*F*_. As one can easily expect, perfect commensurability (i.e., *σ*_1_ = 0) provides the best condition to transfer energy effectively to the IRM, in that the IR is activated at a lower driving force and the energy portion of the IRM is the largest. Comparing Fig. 7a with 7c, one can infer that the modal energy transfer in the 2:1 IR is more sensitively affected by the internal frequency mismatch (*σ*_1_) from the exact 1:2 ratio, while the 1:2 IR shows better robustness regarding the variation in *σ*_1_. Figure 7b, d shows that a larger coupling coefficient *α*_*i*_ is directly related to the threshold driving force for the onset of IR and the amount of energy transferred to the IRM. Contrary to the case of *σ*_1_, *α*_1_ has a more significant impact on the 1:2 IR at its minimum energy level, triggering the IR.

## Discussion

In this paper, we designed and fabricated a geometrically nonlinear, nonprismatic IR system consisting of a silicon microbeam and polymer coupling that incorporates a 1:2 ratio between its second and third mode frequencies. The commensurate relationship between the modes combined with midplane stretching in the nonlinear system realized IR dynamics with strong modal coupling. We successfully characterized the IR experimentally when either the lower or higher mode was driven. We also developed an analytical model for quadratic IR systems based on the energy method for both scenarios when the LM or HM was externally driven. Using this model, we studied the characteristic behaviors of the IR responses while the effective parameters were varied over a range. Finally, we investigated the mechanism of modal energy transfer at different values of the internal frequency mismatch and nonlinear coupling coefficients. Most notably, the analytical model was able to provide valuable insight into the IR mechanism and suggest design strategies to implement IR in a clamped–clamped beam structure: (i) a mid-plane stretching due to the constrained boundary conditions provides the nonlinear (quadratic) coupling mechanism between two flexural modes, which is more dominant than the cubic geometric nonlinearity due to stretching of its own mode, (ii) a higher coupling renders a wider IR dynamic range with a lower activation threshold, (iii) the mode shapes of engaged modes determine the coupling strength, and (iv) coupling the 2nd and 3rd flexural modes in an asymmetric structure is a practically effective method for escalating the IR.

Targeting the desired IR response strongly relies on the accurate allocation of system parameters such that small perturbations in the parameters can drastically alter the activation of IR, nonlinear resonances, and bifurcation points. The current work opens up a new window in which to design quadratic IR systems and understand their complex underlying dynamics. One of the significant findings in this research is that IR can be easily integrated into a simple clamped–clamped beam structure by modifying the geometric parameters to satisfy the IR conditions. Even though the experimental demonstration in this study was performed in a rather complex nonprismatic beam with two dissimilar materials (silicon and polymer), a silicon beam with varying dimensions (e.g., a stepped beam or a tapered beam) can also be employed. Most previous theoretical and experimental studies on IR were based on unconventional structural shapes and modes for M/NEMS applications, which impose more complicated designs for actuation and transduction electrodes. In this regard, a clamped–clamped beam structure that is most commonly used in M/NEMS applications provides a practical platform to derive benefits from the unique dynamic characteristics originating from IR. The strategies suggested in this study can be extended to 2-dimensional plate structures as well. It is also worth mentioning that fixed–fixed strings or membranes with zero flexural rigidity might be alternative candidates to achieve 1:*n* IR, as their mode frequencies inherently entail the commensurability condition^53,54^.

M/NEMSs are great platforms to practically implement IR dynamics due to their flexibility in design and fabrication. In addition, any fabrication randomness can be fairly easily overcome with the frequency tunability of micro/nanoresonators (e.g., applying tension through a gate DC voltage^43,55,56^ or changing the temperature^57,58^). We expect that the feasible implementation of IR in M/NEMSs based on the knowledge obtained in this study can stimulate further research exploiting IR in various applications.

## Materials and methods

### Fabrication

The proposed fabrication sequence started with a silicon-on-insulator (SOI) wafer (Ultrasil Inc.) in which MEMS resonators are commonly fabricated due to the ease of suspending the resonating structures. A 2 μm-thick device layer was patterned using conventional photolithography to delineate an array of silicon microcantilevers. Then, a modified soft lithographic technique, namely, blanket transfer (BT)^59^, was used to transfer a 3 μm-thick photopatternable polyimide (HD4100, HD Microsystem) to the device layer surface from a viscoelastic stamp made of cured polydimethylsiloxane (PDMS). The transferred polyimide film was freestanding over the etched trenches and directly patterned to delineate the polymer microstructures suspended over the gap. The patterned polyimide film was annealed at 350 °C for 3 h under a N_2_ atmosphere. The backside of the SOI wafer was patterned with tight (<5 μm) double-side alignment to open etch windows and etched using deep reactive ion etching (DRIE) until the buried oxide layer was fully exposed. Finally, both the microbeam structure and the polymer freestanding structures were released by hydrofluoric acid etching of the buried oxide layer.

### Experimental process

**Fig. 8:**
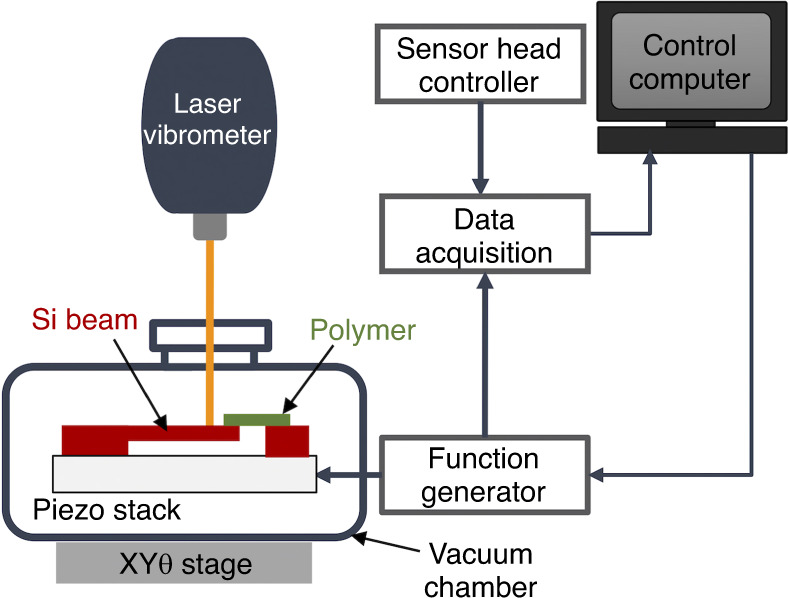
The experimental setup consists of the device actuation, response measurement, and data acquisition/processing sections. The device was driven with a piezoelectric shaker feeding by an AC voltage through a function generator inside a vacuum chamber. Laser Doppler Vibrometry was used to measure the vibratory response, and the obtained signals were sent to a signal processing program through a digital oscilloscope

The experimental setup consisted of device actuation, response measurement, and data acquisition/processing. The experimental setup was carefully designed to eliminate any sources of nonlinearity induced from the actuation and measurement processes, and the observed nonlinearity was deduced to be merely structural. As shown in Fig. 8, the device was driven with a piezoelectric shaker by feeding an AC voltage through a function generator (Tektronix AFG3022c). Laser Doppler vibrometry (Polytec OFV-534 sensor and OFV-5000 controller) was used to measure the dynamic response, and the obtained signals were sent to a signal processing program through a digital oscilloscope (Tektronix DSOX4034A). To capture both the second and third modes simultaneously, the location of the laser was carefully adjusted away from the nodes of these two modes, as shown in Fig. 1a. Because the measurement point does not correspond to a location having a maximum displacement of either mode, only the relative comparison between modal amplitudes is valid. A FFT was performed on the time-domain signals collected at each single-frequency excitation to produce the frequency content of the dynamic response. The FFT-based spectral response revealed all significant peaks that were generated from the nonlinear response, as shown in Fig. 1b, c. The amplitudes and frequencies of these peaks per driving frequency were recorded during the frequency sweep process to obtain the final amplitude responses, as shown in Fig. 2. The experiments were carried out under an absolute vacuum pressure of 3 mTorr to eliminate energy dissipation from air damping.

### Analytical formulation

We employ the energy method to derive the governing equations of the modal amplitude by applying the Euler–Lagrange equation to the Lagrangian of the system. To obtain the energy terms, we start by defining the expression for the transverse displacements of a beam under base excitation. Because both the LM and HM are involved in the IR dynamic responses, two-mode expansion is implemented to describe the transverse displacements (*w*) in the form of:

1 $$w\left( {x,t} \right) = \eta \left[ {A_1\left( t \right)w_1\left( x \right) + A_2\left( t \right)w_2\left( x \right)} \right] + w_b\left( t \right)$$

where *x* is the coordinate of the beam along its length; *t* is time; *A*_1_ and *A*_2_ are the modal amplitudes of the LM and HM, respectively; *w*_1_ and *w*_2_ are the mode shapes of the LM and HM, respectively; and $w_b = - \eta ^2w_F{\mathrm{cos}}\left( {{\mathrm{{\Omega}}}t} \right)$ is the base excitation driving the system at frequency Ω and amplitude *w*_*F*_.

When the system is driven harmonically at a driving frequency of Ω near the LM, the second harmonic motion at 2Ω is internally excited due to the 1:2 IR. Thus, the modal amplitudes *A*_1_ and *A*_2_ are described by:

2 $$\begin{array}{l}A_1\left( t \right) = p_1\left( {\eta t} \right)\cos \left( {\Omega t} \right) + q_1\left( {\eta t} \right)\sin \left( {\Omega t} \right)\\ A_2\left( t \right) = p_2\left( {\eta t} \right)\cos \left( {2\Omega t} \right) + q_2\left( {\eta t} \right)\sin \left( {2\Omega t} \right)\end{array}$$

where $p_i,\,q_i\,\left( {i = 1,\,2} \right)$ are the amplitude components of the modal amplitudes on the slowly varying time scale $\tau = \eta t$. Considering the flexural oscillations in the beam (i.e., with no longitudinal displacement), the nonlinear axial strain-displacement relation is expressed by:

3 $${\it{\epsilon }}_{xx} = - z\frac{{\partial ^2w}}{{\partial x^2}} + \frac{1}{2}\left( {\frac{{\partial w}}{{\partial x}}} \right)^2$$

where *z* is the coordinate along the beam thickness from the neutral plane. Equation (3) includes the strain induced by the linear bending displacement and nonlinear axial stretching during transverse oscillations. Assuming that the only effective strain field in the structure is the axial strain, the strain energy (*U*) for an isotropic material is given by

4 $${\mathrm{d}}U = \left( {\frac{{\upsilon \mu }}{{1 - 2\upsilon }} + \mu } \right){\it{\epsilon }}_{xx}^2$$

where $\mu = E/2\left( {1 + \upsilon } \right)$, in which $\upsilon ,\,E$ are Poisson’s ratio and Young’s modulus, respectively. The strain energy can be calculated by integrating Eq. (4) over the volume of the structure. The kinetic energy is derived by:

5 $$T = \mathop {\int}\limits_0^b {\mathop {\int}\limits_0^h {\mathop {\int}\limits_0^L {\frac{\rho }{2}\dot w^2{\mathrm{d}}V} } }$$

where *ρ* is the density. Then, the Lagrangian is time-averaged over a period of the forcing cycle (from 0 to $\frac{{2\pi }}{{\mathrm{{\Omega}}}}$). The terms up to $O\left( {\eta ^3} \right)$are retained in the averaged Lagrangian.

6 $$\langle {L}\rangle= {\int_0^{\frac{{2\pi }}{{\mathrm{{\Omega}}}}}} {\left( {T - U} \right){\mathrm{d}}t}$$

When the LM is externally driven, the excitation frequency (Ω) is expressed as ${\mathrm{{\Omega}}} = \omega _1 + \eta \sigma _2$, where *σ*_2_ is the external frequency detuning parameter. Substituting Ω into Eq. (6) and using Lagrange’s equation, the following differential equations of the modal amplitudes with respect to the slower time scale are obtained:

7 $$\begin{array}{l}p^{\prime}_1 + \zeta _1p_1 + \sigma _2q_1 + \alpha _1\left( {p_1q_2 - p_2q_1} \right) = 0\\ q^{\prime}_1 + \zeta _1q_1 - \sigma _2p_1 - \alpha _1\left( {p_1p_2 + q_2q_1} \right) + \lambda w_F = 0\\ p^{\prime}_2 + \zeta _2p_2 + \frac{{\omega _2}}{{\omega _1}}\left( {\sigma _2 - \frac{{\sigma _1}}{2}} \right)\,q_2 - 2\alpha _2\left( {p_1q_1} \right) = 0\\ q^{\prime}_2 + \zeta _2q_2 - \frac{{\omega _2}}{{\omega _1}}\left( {\sigma _2 - \frac{{\sigma _1}}{2}} \right)\,p_2 + \alpha _2\left( {p_1^2 - q_1^2} \right) = 0\end{array}$$

where the prime denotes the derivative of variables with respect to *τ*. Here, the modal damping ratios $\left( {\zeta _1,\,\zeta _2} \right)$ are added to the first and second modes. It is worth noting that the nonlinear mode coupling in Eq. (7) originates from the axial strain (*∈*_*xx*_). The axial stretching brings about the cubic coupling terms between the modal amplitudes of *A*_1_ and *A*_2_ (e.g., $A_1^3,A_2^3,A_1^2A_2,\,A_1A_2^2$) in the strain energy, but only the term $A_1^2A_2$ remains as the only effective nonlinear term in the time-averaged Lagrangian equation. After algebraic simplifications, the coefficients of the nonlinear coupling (*α*_1_, *α*_2_) and forcing (*λ*) are given by:

8 $$\begin{array}{l}\alpha _1 = \frac{{{\int}_0^b {{\int}_0^h {{\int}_0^L {\left( {\frac{{\upsilon \mu }}{{1 - 2\upsilon }} + \mu } \right)\left[ {2\left( {w{\prime}_1w{\prime}_2\left( { - zw_1^{\prime \prime }} \right)} \right) + \left( {w{\prime}_1^2\left( { - zw_2^{\prime \prime }} \right)} \right)} \right]{\mathrm{d}}V} } } }}{{4\omega _1{\int}_0^b {{\int}_0^h {{\int}_0^L {\frac{\rho }{2}w_1^2{\mathrm{d}}V} } } }}\\ \alpha _2 = \frac{{{\int}_0^b {{\int}_0^h {{\int}_0^L {\left( {\frac{{\upsilon \mu }}{{1 - 2\upsilon }} + \mu } \right)\left[ {2\left( {w{\prime}_1w_2^\prime \left( { - zw_1^{\prime \prime }} \right)} \right) + \left( {w{\prime}_1^2\left( { - zw_2^{\prime \prime }} \right)} \right)} \right]{\mathrm{d}}V} } } }}{{16\omega _1{\int}_0^b {{\int}_0^h {{\int}_0^L {\frac{\rho }{2}w_2^2{\mathrm{d}}V} } } }}\\ \lambda = \frac{{ - {\int}_0^b {{\int}_0^h {{\int}_0^L {w_1{\mathrm{d}}V} } } }}{{2{\int}_0^b {{\int}_0^h {{\int}_0^L {\frac{\rho }{2}w_1^2{\mathrm{d}}V} } } }}\end{array}$$

The nonlinear coupling coefficients in the first two fractions of Eq. (8) are generated by the pure geometric effect and, thus, determined by the geometric parameters and linear mode shapes of the engaged LM and HM. The numerator of both fractions originates from the identical term $A_1^2A_2$ in the strain energy, and the denominators are derived from the terms in the kinetic energy. Thus, the nonlinear coefficients of *α*_1_ and *α*_2_ are not independent of each other but vary together. The last fraction in Eq. (8) expresses the forcing coefficient, of which the numerator stems from the forcing function and the denominator from the kinetic energy. Equation (8) indicates that the nonlinear and forcing coefficients depend entirely on the mode shapes of the structure. Therefore, one can design 1:2 IR systems with targeted resonance behaviors by tailoring the geometric parameters in Eq. (8).

To obtain the amplitude modulation equation from Eq. (7), the polar transformation is introduced as:

9 $$p_1 = a_1\cos \left( {\beta _1} \right),\,q_1 = a_1\cos \left( {\beta _1} \right)\,p_2 = a_2\cos \left( {\beta _2} \right),\,q_2 = a_2{\mathrm{sin}}\left( {\beta _2} \right)$$

Then, the equations describing the amplitude and phase modulation are obtained as:

10 $$\begin{array}{l}a{\prime}_1=-\zeta_1a_1+\alpha_1a_1a_2\sin\left({2\beta_1-\beta_2}\right)- {\mathrm{{\Lambda}}}\sin\left({\beta _1}\right)\\ a_1\beta{\prime}_1=\sigma_2a_1+\alpha _1a_1a_2\cos\left({2\beta_1-\beta_2}\right)-{\mathrm{{\Lambda}}}\cos\left({\beta_1}\right)\\ a{\prime}_2=-\zeta_2a_2+\alpha _2a_1^2\sin\left( {2\beta_1-\beta_2}\right)\\a_2\beta{\prime}_2=\frac{{\omega_2}}{{\omega_1}}\left({\sigma_2-\frac{{\sigma_1}}{2}}\right)a_2-\alpha_2a_1^2\cos\left({2\beta_1-\beta_2}\right)\end{array}$$

where ${\Lambda} = \lambda w_F$. With $a^{\prime}_1 = a^{\prime}_2 = \beta ^{\prime}_1 = \beta ^{\prime}_2 = 0$, the coupled algebraic equations for the analytical steady-state amplitudes (*a*_1_, *a*_2_) and phases (*β*_1_, *β*_2_) can be obtained as:

11 $$\begin{array}{l}\left({\zeta_2^2 +\left({\frac{{\omega_2}}{{\omega_1}}\left({\sigma_2- 0.5\sigma_1}\right)}\right)^2}\right)a_2^2=\alpha_2^2a_1^4\\\frac{{\alpha_1^2\alpha_2^2}}{{\zeta_2^2 +\left({\frac{{\omega_2}}{{\omega_1}}\left({\sigma_2-0.5\sigma_1}\right)} \right)^2}}a_1^6+\frac{{2\alpha_1\alpha_2\left({-\zeta_1\zeta_2+\frac{{\omega_2}}{{\omega _1}}\sigma_2\left({\sigma_2-0.5\sigma_1}\right)}\right)}}{{\zeta_2^2 +\left({\frac{{\omega _2}}{{\omega_1}}(\sigma_2-0.5\sigma_1)} \right)^2}}a_1^4+\left({\zeta_1^2 +\sigma_2^2} \right)a_1^2-{\Lambda}^2=0\\\tan\beta_1=\frac{{\omega_1\left({-\zeta_1\alpha_2a_1^2+\alpha _1\zeta_2a_2^2}\right)}}{{\omega_1\sigma_2\alpha_2a_1^2+\alpha_1\omega_2\left({\sigma_2- 0.5\sigma_1}\right)a_2^2}}\\\tan\beta_2=\frac{{-\zeta_2\omega_1\left({1-\tan^2\beta_1} \right)+2\omega_2{\mathrm{tan}}\beta_1\left({\sigma_2-0.5\sigma_1}\right)}}{{\omega_2\left( {\sigma_2-0.5\sigma_1}\right)\left({1-\tan^2\beta_1}\right)+2\zeta_2\tan\beta_1\omega _1}}\end{array}$$

The solutions to Eq. (11) define the steady-state amplitudes and phases of the system. From Eq. (11), one can see that there exist one or three real solutions for *a*_1_, and for each solution of *a*_1_, a corresponding *a*_2_ always exists (the two being a pair). The stability of a fixed point obtained by Eq. (11) is evaluated by calculating the eigenvalues of the Jacobian linearization matrix of Eq. (10), which is given by

12 $$J=\left({\begin{array}{*{20}{c}}{-\zeta_1+\alpha_1a_2{\mathrm{sin}}\left({2\beta_1-\beta_2} \right)}&{2\alpha_1a_1a_2\cos\left({2\beta_1-\beta_2}\right)-{\Lambda}{\mathrm{cos}}\left( {\beta_1}\right)}&{\alpha_!a_1{\mathrm{sin}}\left({2\beta_1-\beta_2}\right)}&{-\alpha _1a_1a_2{\mathrm{cos}}\left({2\beta_1-\beta_2}\right)}\\{\frac{{\Lambda}}{{a_1^2}}{\mathrm{cos}}\left({\beta_1}\right)}&{-2\alpha_1a_2\sin \left({2\beta_1-\beta_2}\right)+ \frac{{\Lambda}}{{a_1}}{\mathrm{sin}}\left( {\beta _1} \right)}&{\alpha_1\cos\left({2\beta_1-\beta_2}\right)}&{\alpha_1a_2\sin\left({2\beta_1-\beta_2}\right)}\\ {2\alpha _2a_1\sin\left({2\beta_1-\beta_2}\right)\left({2\beta_1-\beta_2}\right)}&{2\alpha_2a_1^2\cos \left({2\beta_1-\beta_2}\right)}&{-\zeta_2}&{-\alpha_2a_1^2\cos\left({2\beta_1-\beta_2} \right)}\\{-\frac{{2\alpha_2a_1}}{{a_2}}\cos\left({2\beta_1-\beta_2}\right)}&{\frac{{2\alpha_2a_1^2}}{{a_2}}\sin\left( {2\beta_1-\beta_2}\right)}&{\frac{{\alpha_2a_1^2}}{{a_2}}\cos\left({2\beta_1-\beta_2}\right)}&{-\frac{{\alpha_2a_1^2}}{{a_2}}\sin\left({2\beta_1-\beta_2}\right)}\end{array}}\right)$$

If all of the eigenvalues at the equilibrium point have negative real parts, the point is asymptotically stable. Otherwise, the solution is unstable.

When the HM is externally driven, the excitation frequency (Ω) is expressed as ${\mathrm{{\Omega}}} = \omega _2 + \eta \sigma _2$, and the 2:1 IR excites the LM at the frequency Ω/2. Thus, the expressions for the modal amplitudes change from Eq. (2) to

13 $$\begin{array}{l}A_1\left(t\right)=p_1\left({\eta t}\right)\cos\left({\frac{\Omega}{2}t}\right)+ q_1\left({\eta t}\right)\sin\left({\frac{\Omega}{2}t}\right)\\A_2\left(t\right)=p_2\left({\eta t}\right)\cos\left({\Omega t}\right)+ q_2\left({\eta t}\right)\sin\left({\Omega t}\right)\end{array}$$

Then, we follow the same procedure to investigate the 2:1 IR. The detailed derivation is provided in the Supplementary Information. The analytical expressions for the steady-state amplitudes and phases are given by:

14 $$\begin{array}{l}a_2^2=\frac{{\zeta_1^2+\left({\frac{{\sigma_1+\sigma_2}}{2}}\right)^2}}{{\overline{\alpha_1} ^2}}\\\left({\overline{\alpha_1}\overline{\alpha_2}}\right)^2a_1^4+ \left({-2\overline{\alpha_1}\overline{\alpha_2}\left({\zeta_1\zeta_2-\sigma_2\left( {\frac{{\sigma_1+\sigma_2}}{2}}\right)}\right)}\right)a_1^2+\left({\left({\zeta_2^2+\sigma _2^2}\right)\left({\zeta_1^2+\left({\frac{{\sigma_1+\sigma_2}}{2}}\right)^2}\right)-\overline {\alpha_1}^2\bar{\Lambda}^2}\right)=0\\\tan\beta_2=\frac{{\overline {\alpha_1} \zeta _2a_2^2+\overline{\alpha_2}\zeta_1a_1^2}}{{\overline{\alpha_2}\left({\frac{{\sigma_1 + \sigma_2}}{2}}\right)a_1^2-\sigma_2\overline{\alpha_1}a_2^2}}\\\left({\frac{{2\zeta _1}}{{(\sigma_1+\sigma_2)}}+\tan\beta_2}\right)\tan^2\beta_1+\left({2-\frac{{4\zeta_1\tan \beta_2}}{{\sigma_1+\sigma_2}}}\right)\tan\beta_1-\left({\frac{{2\zeta_1}}{{(\sigma_1 + \sigma_2)}}+\tan\beta_2}\right)=0\end{array}$$

The solutions to Eq. (14) define the steady-state amplitudes and phases of the 2:1 IR response when the HM is externally driven. From the amplitude equations, we can see that there always exists one real solution for the ERM (i.e., HM) amplitude of *a*_2_, while no or two real solutions exist for the IRM (i.e., LM) amplitude of *a*_1_.

It is worth noting that the model we developed for a quadratic IR system does not include all the nonlinear terms associated with the quadratic modal coupling terms in Eq. (10) and Eq. (S7). The general form of the equations of motion for a nonlinear system with quadratic coupling terms is expressed by:

15 $$\begin{array}{l}\ddot u_1 + \omega _1^2u_1 + \eta \left\{ {2\zeta _1\dot u_1 + \gamma _1u_1^2 + \gamma _2u_1u_2 + \gamma _3u_2^2 - f_1{\mathrm{cos}}\left( {{\mathrm{{\Omega}}}t} \right)} \right\} = 0\\ \ddot u_2 + \omega _2^2u_2 + \eta \left\{ {2\zeta _2\dot u_1 + \gamma _4u_1^2 + \gamma _5u_1u_2 + \gamma _6u_2^2 - f_2{\mathrm{cos}}({\mathrm{{\Omega}}}t)} \right\} = 0\end{array}$$

When we impose the condition of commensurability by introducing equation $\omega _2 = 2\omega _1 + \eta \sigma _1$ and drive the system around its lower mode ${\mathrm{{\Omega}}} = \omega _1 + \eta \sigma _2$, Eq. (15) can be reduced to obtain the amplitude-modulated equations using the method of multiple scales:

16 $$\begin{array}{lll}a_{1}^{\prime} = -\zeta_1a_1+\gamma_2a_1a_2 \sin\beta_2 + \Lambda \sin\beta_1a_1\beta_{1}^{\prime}=\omega_1 \sigma_2 a_1-\gamma_2a_1a_2\cos\beta_2+\Lambda\cos\beta_1a_{2}^{\prime}=-\zeta_2a_2-\gamma_4a_1^2\sin\beta_2a_2\beta_{2}^{\prime}=\omega_2(\sigma_2-\frac{\sigma_1}{2})a_2-\gamma_4a_1^2\cos\beta_2\end{array}$$

Equation (16) reveals that the only effective coupling coefficients in the system are *γ*_2_ and *γ*_4_, with none of the remaining nonlinear coefficients appearing in the final leading-order solution. These equations, which are consistent with the ones provided by Nayfeh^5,60^, corroborate that our energy-based model is general for a quadratic IR system and is not limited to specific nonlinear coupling terms.

## Supplementary information

Supplemental Material
