# Supplementary material for: Strong internal resonance in a nonlinear, asymmetric microbeam resonator
Source: Microsyst Nanoeng. 2021 Jan 26;7:9. doi: 10.1038/s41378-020-00230-1 (PMC8433341; doi:10.1038/s41378-020-00230-1)
Supplement: Supplementary file 1 — Supplemental Material [file 41378_2020_230_MOESM1_ESM.docx]

**Supplementary Information**

**Strong Internal Resonance in a Nonlinear, Asymmetric Micromechanical Resonator**

Keivan Asadi^1^, Junghoon Yeom^2^, Hanna Cho^1,*^

^1^Dept. of Mechanical and Aerospace Engineering, The Ohio State University, Columbus, Ohio, USA

^2^Dept. of Mechanical Engineering, Michigan State University, East Lansing, Michigan, USA

^*^Corresponding author: Hanna Cho, [cho.867@osu.edu](mailto:cho.867@osu.edu)

1. **Derivation of the nonlinear intermodal coupling coefficient in 1:2 Internal Resonance**

Substituting Eq. (1) and Eq. (3) into Eq. (4) and integrating over the volume, the strain energy can be expanded up to$O\left( \eta^{3} \right)$

| $U=\iiint\left( \frac{\upsilon\mu}{1-2\upsilon}+\mu\right)\eta^{2}\{A_{1}^{2}\left( z^{2}w_{1}^{''2} \right)+A_{2}^{2}\left( z^{2}w_{2}^{''2} \right)+2A_{1}A_{2}z^{2}w_{1}^{''}w_{2}^{''}-{\eta A}_{1}^{3}zw_{1}^{'2}w_{1}^{''}-\eta A_{1}^{2}A_{2}\left( 2zw_{1}^{'}w_{2}^{'}w_{1}^{''}+zw_{1}^{'2}w_{2}^{''} \right)-{\eta A}_{1}A_{2}^{2}\left( 2zw_{1}^{'}w_{2}^{'}w_{2}^{''}+zw_{2}^{'2}w_{1}^{''} \right)-{\eta A}_{2}^{3}(zw_{2}^{'2}w_{2}^{''})\}dV$ | (S1) |
| --- | --- |

The quadratic and cubic terms in the strain energy correspond to linear bending and nonlinear stretching energies, respectively. Modal amplitude equations in Eq. (2) are plugged into Eq. (S1) and then Eq. (S1) is integrated over a period of forcing cycle to obtain time-averaged strain energy. Through the time integration process, as mentioned in the manuscript, all nonlinear terms but the term $A_{1}^{2}A_{2}$ become zero. The time-averaged strain energy is written as

| $\left\langle U \right\rangle=\int_{0}^{\frac{2\pi}{\Omega}} Udt$  $=\frac{1}{\Omega}\eta^{2}\left( \frac{\upsilon\mu}{1-2\upsilon}+\mu\right)\left[ \{\pi(p_{1}^{2}+q_{1}^{2})\iiint\left( z^{2}w_{1}^{''2} \right)dV\}+\{\pi\left( p_{2}^{2}+q_{2}^{2} \right)\iiint\left( z^{2}w_{2}^{''2} \right)dV\}-\{\frac{\pi(p_{1}^{2}p_{2}-p_{2}q_{1}^{2}+2p_{1}q_{1}q_{2})}{2}\iiint\left( 2zw_{1}^{'}w_{2}^{'}w_{1}^{''}+zw_{1}^{'2}w_{2}^{''} \right)dV\} \right]$ | (S2) |
| --- | --- |

where the last term is responsible for the nonlinear intermodal coupling effect in the system. Similarly, by substituting Eq. (1)-(2) into Eq. (5) and integrating over time, one can write the time-averaged kinetic energy up to$O\left( \eta^{3} \right)$

| $\left\langle T \right\rangle=\int_{0}^{\frac{2\pi}{\Omega}} Tdt$  $=\eta^{2}\left[ \{\pi\Omega(p_{1}^{2}+q_{1}^{2})\iiint\left( \frac{\rho}{2}w_{1}^{2} \right)dV\}+\{4\pi\Omega\left( p_{2}^{2}+q_{2}^{2} \right)\iiint\left( \frac{\rho}{2}w_{2}^{2} \right)dV\} \right]$  $+\eta^{3}\left[ \{-\pi w_{F}p_{1}\iiint\left( \frac{\rho}{2}w_{1} \right)dV\}+\{2\pi\left( p_{1}^{'}q_{1}-p_{1}q_{1}^{'} \right)\iiint\left( \frac{\rho}{2}w_{1}^{2} \right)dV\}+\{4\pi\left( p_{2}^{'}q_{2}-p_{2}q_{2}^{'} \right)\iiint\left( \frac{\rho}{2}w_{2}^{2} \right)dV\} \right]$ | (S3) |
| --- | --- |

After obtaining the averaged Lagrangian $\left\langle L \right\rangle=\left\langle T-U \right\rangle$ and imposing$\Omega=\omega_{1}+\eta\sigma_{2}, \omega_{2}=2\omega_{1}+\eta\sigma_{1}$, one can use Euler-Lagrange equation $\left( \frac{\partial\left\langle L \right\rangle}{\partial p_{i}'} \right)^{'}-\frac{\partial\left\langle L \right\rangle}{\partial p_{i}}=0, \left( \frac{\partial\left\langle L \right\rangle}{\partial q_{i}'} \right)^{'}-\frac{\partial\left\langle L \right\rangle}{\partial q_{i}}=0, i=1,2$ to obtain differential equations for modal amplitudes in Eq. (7). This reveals the equations for the nonlinear modal coupling equations expressed in Eq. (8).

1. **Intermodal coupling coefficients depending on the mode shape**

We extend the calculation of nonlinear coefficients in a 1:2 IR system to include more examples. First, we consider the symmetrical and asymmetrical mode shapes same as the ones in the main manuscript and extend the calculation up to the fifth mode. Figure S1 shows the symmetrical mode shapes with $w_{n}\left( x \right)=\sin\left( \frac{n\pi}{L}x \right)$ and asymmetrical mode shapes with $w_{n}\left( x \right)=\sin\left( \frac{n\pi}{L}x^{2} \right)$ where $n=1, 2,\ldots, 5$. The computed nonlinear coupling coefficients using Eq. (8) for these configurations are brought in Tables S1. These are the extension of the results in Table I to 4^th^ and 5^th^ modes. These results confirm that asymmetric mode shapes generally have higher coupling coefficients. When HM is an even number mode (n=2,4) in the symmetric case, the nonlinear coefficient becomes zero and IR cannot be activated. The results also indicate that combinations of higher modes can induce even stronger intermodal coupling. In practice, however, it is quite difficult to experimentally realize flexural modes higher than the third mode and, thus, coupling 2nd and 3rd mode could be considered as a practically viable option.


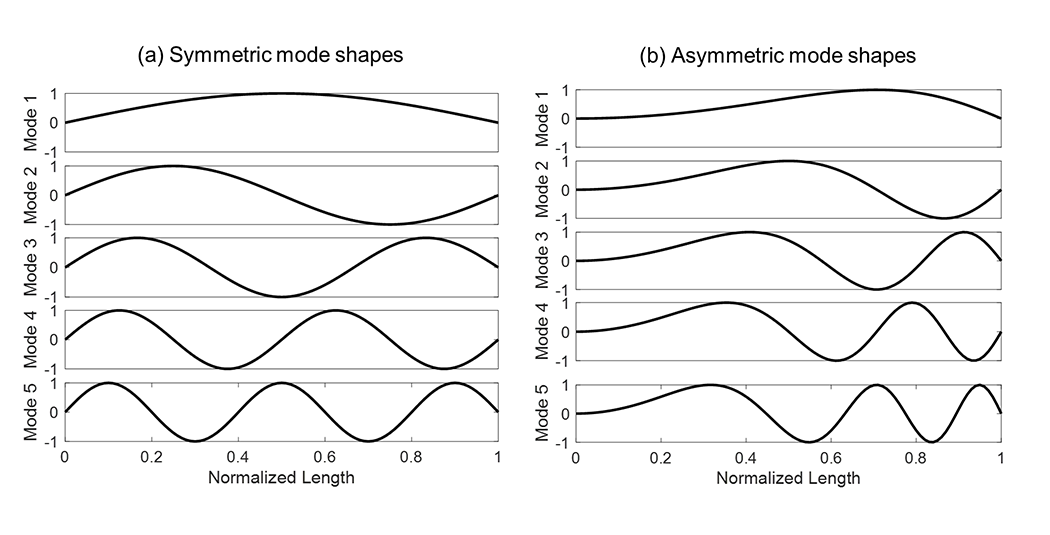

**Figure S1:** First five flexural mode shapes with (a) symmetrical configuration $w_{n}\left( x \right)=\sin\left( \frac{n\pi}{L}x \right)$ and (b) asymmetrical configuration with $w_{n}\left( x \right)=\sin\left( \frac{n\pi}{L}x^{2} \right)$for $n=1, 2,\ldots,5$.

**Table S1.** Nonlinear coefficients in 1:2 IR in a microbeam with zero displacement boundary conditions with symmetrical,$w_{n}\left( x \right)=\left( w_{n}\left( x \right)=\sin\left( \frac{n\pi}{L}x \right) \right)$, and asymmetrical$\left( w_{n}\left( x \right)=\sin\left( \frac{n\pi}{L}x^{2} \right) \right)$ flexural modes. Geometric parameters other than the mode shapes are set to be constant as $\rho=1, \left( \frac{\upsilon\mu}{1-2\upsilon}+\mu\right)=1, L=1, b=0.1, h=0.01$. The first column indicates the LM number, and the second row indicates the HM number.

| $\boldsymbol{\vert}\boldsymbol{\alpha}_{\boldsymbol{1}}\boldsymbol{\vert}$($\boldsymbol{\vert\alpha}_{\boldsymbol{2}}\boldsymbol{\vert}$) | Symmetric mode shapes | | | | Asymmetric mode shapes | | | |
| --- | --- | --- | --- | --- | --- | --- | --- | --- |
|  | Mode 2 | Mode 3 | Mode 4 | Mode 5 | Mode 2 | Mode 3 | Mode 4 | Mode 5 |
| Mode 1 | **0.0(0.0)** | **2.2(0.6)** | **0.0(0.0)** | **3.3(0.8)** | **5.7(1.3)** | **10.9(2.4)** | **14.4(3.1)** | **17.0(3.6)** |
| Mode 2 |  | **3.2(0.8)** | **0.0(0.0)** | **17.9(4.5)** |  | **18.8(4.5)** | **41.2(9.7)** | **78.2(18.2)** |
| Mode 3 |  |  | **0.0(0.0)** | **5.0(1.3)** |  |  | **56.4(13.8)** | **44.2(10.7)** |
| Mode 4 |  |  |  | **29.3(7.3)** |  |  |  | **135.3(33.3)** |

We performed same analysis in a doubly clamped beam (zero displacement and slope) in both symmetric and asymmetric mode shapes. Mode shapes of a uniform c-c are expressed by$w_{n}\left( x \right)=\left( \cos\beta_{n}x-\cosh\beta_{n}x \right)-\frac{\cos\beta_{n}L-\cosh\beta_{n}L}{\sin\beta_{n}L-\sin\beta_{n}L}(\sin\beta_{n}x-\sinh\beta_{n}x)$ where$\beta_{1}L=4.73, \beta_{2}L=7.85,\beta_{3}L=10.99, \beta_{4}L=14.14, \beta_{5}L=17.28$ (see Fig. S2(a)). For asymmetric mode shapes, we assumed the trial functions take the form of$w_{n}\left( x \right)=\left( \cos\beta_{n}x^{2}-\cosh\beta_{n}x^{2} \right)-\frac{\cos\beta_{n}L-\cosh\beta_{n}L}{\sin\beta_{n}L-\sin\beta_{n}L}(\sin\beta_{n}x^{2}-\sinh\beta_{n}x^{2})$. The asymmetric mode shapes plotted in Fig. S2(b) are analogous to modes of the microbeam studied in the paper. Table S2 shows the values for modal coupling coefficients between combinations of the first five modes in the symmetric and asymmetric doubly clamped beams. The variation trend of nonlinear coefficients with the modes is fairly similar to the first study.


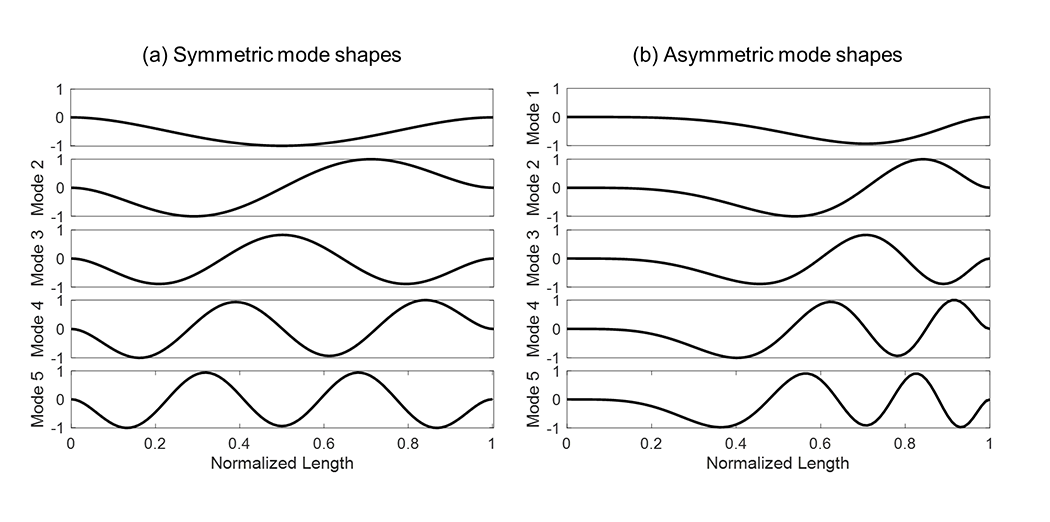

**Figure S2:** First five flexural mode shapes of a doubly clamped (a) symmetric and (b) asymmetric beam.

**Table S2.** Nonlinear coefficients in 1:2 IR in a doubly clamped microbeam with symmetrical and asymmetricalflexural modes. Geometric parameters other than the mode shapes are set to be constant as $\rho=1, \left( \frac{\upsilon\mu}{1-2\upsilon}+\mu\right)=1, L=1, b=0.1, h=0.01$.

| $\boldsymbol{\vert}\boldsymbol{\alpha}_{\boldsymbol{1}}\boldsymbol{\vert}$($\boldsymbol{\vert\alpha}_{\boldsymbol{2}}\boldsymbol{\vert}$) | Symmetric mode shapes | | | | Asymmetric mode shapes | | | |
| --- | --- | --- | --- | --- | --- | --- | --- | --- |
|  | Mode 2 | Mode 3 | Mode 4 | Mode 5 | Mode 2 | Mode 3 | Mode 4 | Mode 5 |
| Mode 1 | **0.0(0.0)** | **2.5(0.6)** | **0.0(0.0)** | **0.4(0.1)** | **3.5(0.8)** | **10.9(2.5)** | **8.4(1.8)** | **0.2(0.0)** |
| Mode 2 |  | **5.2(1.3)** | **0.0(0.0)** | **22.0(5.5)** |  | **17.3(4.2)** | **18.5(4.3)** | **87.5(20.1)** |
| Mode 3 |  |  | **2.4(0.6)** | **26.6(6.7)** |  |  | **28.3(6.9)** | **88.4(21.2)** |
| Mode 4 |  |  |  | **13.0(3.2)** |  |  |  | **50.7(12.4)** |

1. **2:1 Internal Resonance**

When the HM is externally driven, the excitation frequency ($\Omega)$ is expressed as$\Omega=\omega_{2}+\eta\sigma_{2}$ and the 2:1 IR excites the LM at the frequency $\Omega/2$. Thus, the expression of modal amplitudes and corresponding differential equations change from Eq. (2) and Eq. (7) into

$A_{1}\left( t \right)=p_{1}\left( \eta t \right)\cos\left( \frac{Ω}{2}t \right)+q_{1}\left( \eta t \right)\sin\left( \frac{Ω}{2}t \right)$ (S4)

$A_{2}\left( t \right)=p_{2}\left( \eta t \right)\cos\left( Ωt \right)+q_{2}\left( \eta t \right)\sin\left( Ωt \right)$.

and

| $p_{1}^{'}+\zeta_{1}p_{1}+\frac{\omega_{1}}{\omega_{2}}(\sigma_{1}+\sigma_{2})q_{1}+\bar{\alpha_{1}}\left( p_{1}q_{2}-p_{2}q_{1} \right)=0$  $q_{1}^{'}+\zeta_{1}q_{1}-\frac{\omega_{1}}{\omega_{2}}(\sigma_{1}+\sigma_{2})p_{1}-\bar{\alpha_{1}}\left( p_{1}p_{2}+q_{2}q_{1} \right)=0$  $p_{2}^{'}+\zeta_{2}p_{2}+\sigma_{2}q_{2}-2\bar{\alpha_{2}}\left( p_{1}q_{1} \right)=0$  $q_{2}^{'}+\zeta_{2}q_{2}-\sigma_{2} p_{2}+\bar{\alpha_{2}}\left( p_{1}^{2}-q_{1}^{2} \right)+\bar{\lambda}w_{F}=0$ | (S5) |
| --- | --- |

where the nonlinear coefficients$\left( \bar{\alpha_{1}},\bar{\alpha_{2}} \right)$and $\bar{\lambda}$ are given by:

| $\bar{\alpha_{1}}=\frac{\int_{0}^{b} \int_{0}^{h} \int_{0}^{L} (\frac{\upsilon\mu}{1-2\upsilon}+\mu)\left[ 2\left( w_{1}^{'}w_{2}'\left( -zw_{1}'' \right) \right)+\left( w_{1}^{'2}\left( -zw_{2}'' \right) \right) \right]dV}{2\omega_{2}\int_{0}^{b} \int_{0}^{h} \int_{0}^{L} \frac{\rho}{2}w_{1}^{2}dV}$  $\bar{\alpha_{2}}=\frac{\int_{0}^{b} \int_{0}^{h} \int_{0}^{L} (\frac{\upsilon\mu}{1-2\upsilon}+\mu)\left[ 2\left( w_{1}^{'}w_{2}'\left( -zw_{1}'' \right) \right)+\left( w_{1}^{'2}\left( -zw_{2}'' \right) \right) \right]dV}{8\omega_{2}\int_{0}^{b} \int_{0}^{h} \int_{0}^{L} \frac{\rho}{2}w_{2}^{2}dV}$  $\bar{\lambda}=\frac{-\int_{0}^{b} \int_{0}^{h} \int_{0}^{L} w_{2}dV}{\int_{0}^{b} \int_{0}^{h} \int_{0}^{L} \frac{\rho}{2}w_{2}^{2}dV}$ | (S6) |
| --- | --- |

Note that the relations$\bar{\alpha_{1}}=2\alpha_{1}$, $\bar{\alpha_{2}}=2\alpha_{2}$ and $\bar{\lambda}=2\lambda$exist between the LME and HME cases. Using the polar transformation in Eq. (9), the amplitude and phase modulation equations are obtained as Eq. (S7).

| $a_{1}^{'}=-\zeta_{1}a_{1}+\bar{\alpha_{1}}a_{1}a_{2}\sin\left( 2\beta_{1}-\beta_{2} \right)$  $a_{1}\beta_{1}^{'}=\left( \frac{\sigma_{1}+\sigma_{2}}{2} \right)a_{1}+\bar{\alpha_{1}}a_{1}a_{2}\cos\left( 2\beta_{1}-\beta_{2} \right)$  $a_{2}^{'}=-\zeta_{2}a_{2}+\bar{\alpha_{2}}a_{1}^{2}\sin\left( \beta_{2}-2\beta_{1} \right)-\bar{\Lambda}\sin\left( \beta_{2} \right)$  $a_{2}\beta_{2}^{'}=\sigma_{2}a_{2}+\bar{\alpha_{2}}a_{1}^{2}\cos\left( \beta_{2}-2\beta_{1} \right)-\bar{\Lambda}cos(\beta_{2})$ | (S7) |
| --- | --- |

where$\bar{\Lambda}=\bar{\lambda}w_{F}$. The analytical expressions for the steady state amplitudes and phases are found as following:

| $a_{2}^{2}=\frac{\zeta_{1}^{2}+\left( \frac{\sigma_{1}+\sigma_{2}}{2} \right)^{2}}{{\bar{\alpha_{1}}}^{2}}$  $\left( \bar{\alpha_{1}}\bar{\alpha_{2}} \right)^{2}a_{1}^{4}+\left( -2\bar{\alpha_{1}}\bar{\alpha_{2}}\left( \zeta_{1}\zeta_{2}-\sigma_{2}\left( \frac{\sigma_{1}+\sigma_{2}}{2} \right) \right) \right)a_{1}^{2}+\left( \left( \zeta_{2}^{2}+\sigma_{2}^{2} \right)\left( \zeta_{1}^{2}+\left( \frac{\sigma_{1}+\sigma_{2}}{2} \right)^{2} \right)-{\bar{\alpha_{1}}}^{2}\bar{\Lambda}^{2} \right)=0$  $\tan\beta_{2}=\frac{\bar{\alpha_{1}}\zeta_{2}a_{2}^{2}+\bar{\alpha_{2}}\zeta_{1}a_{1}^{2}}{\bar{\alpha_{2}}\left( \frac{\sigma_{1}+\sigma_{2}}{2} \right)a_{1}^{2}-\sigma_{2}\bar{\alpha_{1}}a_{2}^{2}}$  $\left( \frac{2\zeta_{1}}{{(\sigma}_{1}+\sigma_{2})}+tan\beta_{2} \right)\tan^{2} \beta_{1}+\left( 2-\frac{4\zeta_{1}\tan\beta_{2}}{\sigma_{1}+\sigma_{2}} \right)\tan\beta_{1}-\left( \frac{2\zeta_{1}}{{(\sigma}_{1}+\sigma_{2})}+\tan\beta_{2} \right)=0$ | (S8) |
| --- | --- |

The solutions of Eq. (S8) define the steady-state amplitudes and phases of the 2:1 IR response. From the amplitude equations, we can see that there always exists one real solution for the ERM amplitude of $a_{2},$ while up to two real solutions can exist for the IRM amplitude of$a_{1}$. The eigenvalue analysis of the Jacobian matrix at the fixed points in Eq. (S8) determines the stability of those solutions. The Jacobian matrix is derived from Eq. (S7):

| $\bar{J}=\left( \begin{matrix} -\zeta_{1}+\bar{\alpha_{1}}a_{2}sin(2\beta_{1}-\beta_{2}) & 2\bar{\alpha_{1}}{a_{1}a}_{2}\cos\left( 2\beta_{1}-\beta_{2} \right) & \bar{\alpha_{1}}a_{1}sin(2\beta_{1}-\beta_{2}) & -\bar{\alpha_{1}}a_{1}a_{2}\cos(2\beta_{1}-\beta_{2}) \\ 0 & -2\bar{\alpha_{1}}a_{2}\sin(2\beta_{1}-\beta_{2}) & \bar{\alpha_{1}}\cos(2\beta_{1}-\beta_{2}) & \bar{\alpha_{1}}a_{2}\sin(2\beta_{1}-\beta_{2}) \\ 2\bar{\alpha_{2}}a_{1}\sin(\beta_{2}-2\beta_{1}) & -2\bar{\alpha_{2}}a_{1}^{2}\cos(\beta_{2}-2\beta_{1}) & -\zeta_{2} & \bar{\alpha_{2}}a_{1}^{2}cos(\beta_{2}-2\beta_{1})-\bar{\Lambda}\cos\left( \beta_{2} \right) \\ \frac{2\bar{\alpha_{2}}a_{1}}{a_{2}}\cos(\beta_{2}-2\beta_{1}) & \frac{2\bar{\alpha_{2}}a_{1}^{2}}{a_{2}}\sin(\beta_{2}-2\beta_{1}) & -\frac{\bar{\alpha_{2}}a_{1}^{2}}{a_{2}^{2}}\cos\left( \beta_{2}-2\beta_{1} \right)+\frac{\bar{\Lambda}}{a_{2}^{2}}cos(\beta_{2}) & -\frac{\bar{\alpha_{2}}a_{1}^{2}}{a_{2}}\sin\left( \beta_{2}-2\beta_{1} \right)++\frac{\bar{\Lambda}}{a_{2}}sin(\beta_{2}) \end{matrix} \right)$ | (S9) |
| --- | --- |

1. **Quasi-periodic modal responses in 1:2 IR system**

The ERM and IRM amplitude responses become unstable in a portion of the single solution branch near the center dip of the M-shaped plots in 1:2 IR systems. In this instability region, no stable steady-state solution exists in the dynamic response but the quasi-periodic resonant response happens to appear. We numerically simulated the ERM and IRM time responses in Eq. (15) with the appropriate parameter values generating the unstable single solution branch. Figure S3, showing an excerpt of the resulting time series simulation of ERM and IRM responses, clearly demonstrate the continuous energy exchange between these two modes to generate the quasi-periodic response.


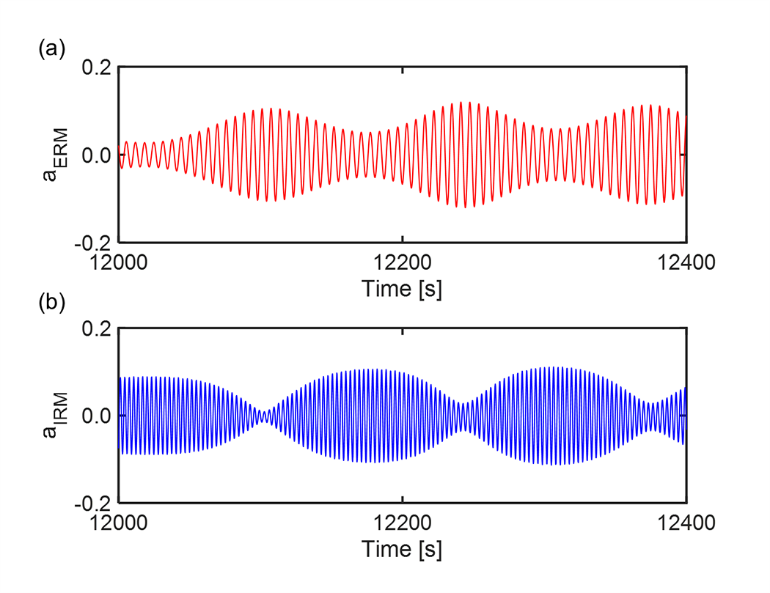


**Figure S3:** Quasi-periodic time series response of ERM and IRM in 1:2 IR system for${(\omega}_{1}=1, \omega_{2}=2, \zeta_{1}=\zeta_{2}=0.001, {|\gamma}_{2}\left| =1, {|\gamma}_{4} \right|=2, f_{1}=f_{2}=7e-4,{\sigma_{1}=0,\sigma}_{2}=-0.01$.

1. **Parametric study of IR amplitude responses**

Figures S4-S6 show a closer view of the ERM and IRM responses by rendering the 3D graphs in Figures 5-6 on a 2-dimensional amplitude-frequency planes with varying values of the base excitation level ($w_{F}$), internal detuning parameter ($\sigma_{1}$), and nonlinear coefficients ($\alpha_{i}$), respectively. The left and right columns in Figures S4-S6 correspond to 1:2 and 2:1 IR, respectively.


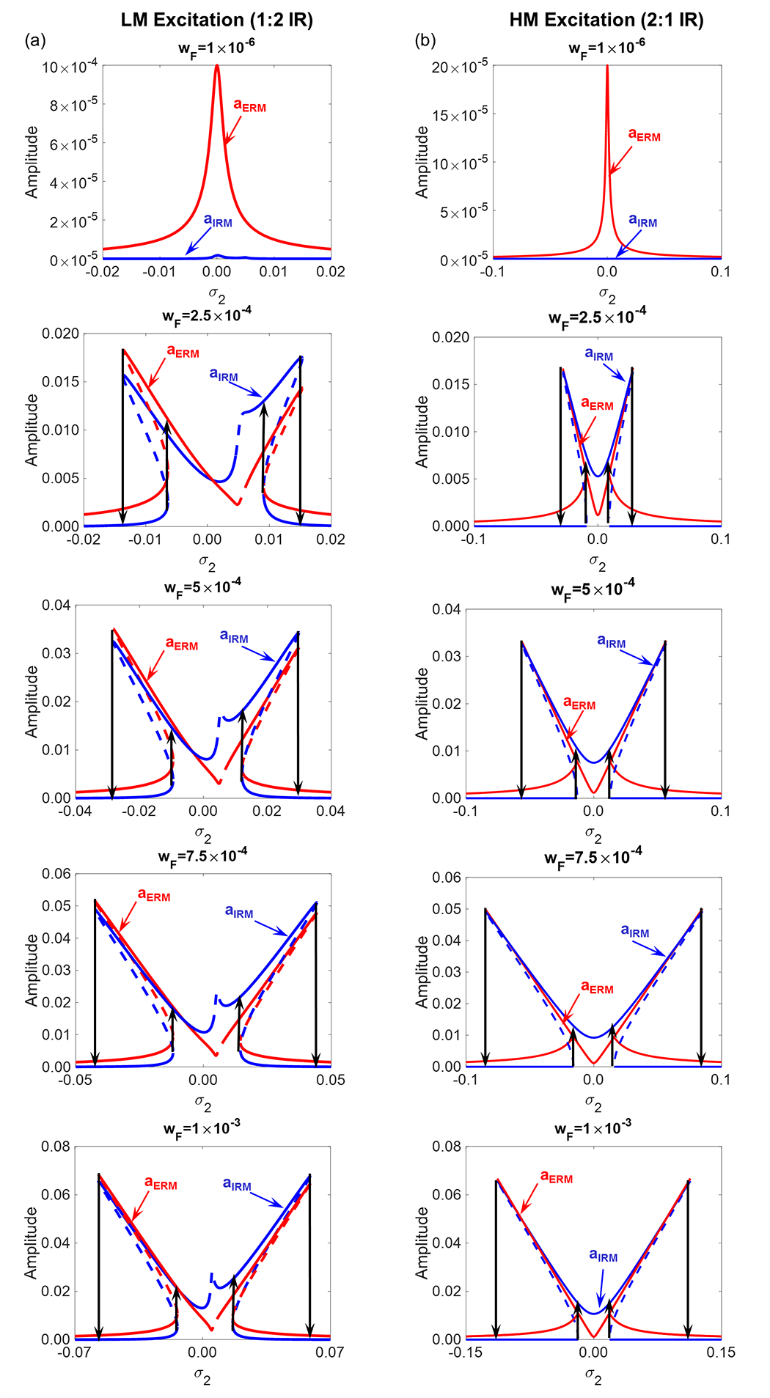


**Figure S4:** (a) Parametric study of the IR amplitude responses in a 1:2 IR system in varying base driving levels with system parameter values of$\omega_{1}=1, \omega_{2}=2, \zeta_{1}=\zeta_{2}=0.001, {|\alpha}_{1}|=0.87, |\alpha_{2}|=1.75, \lambda=0.1,\sigma_{1}=0.01$. (b) Parametric study of the IR amplitude responses in a 2:1 IR system in varying base driving levels with system parameter values of $\omega_{1}=1, \omega_{2}=2, \zeta_{1}=\zeta_{2}=0.001,| \bar{\alpha}_{1}|=0.85,| \bar{\alpha}_{2}|=1.75, {\bar{\lambda}=0.2,\sigma}_{1}=0$.


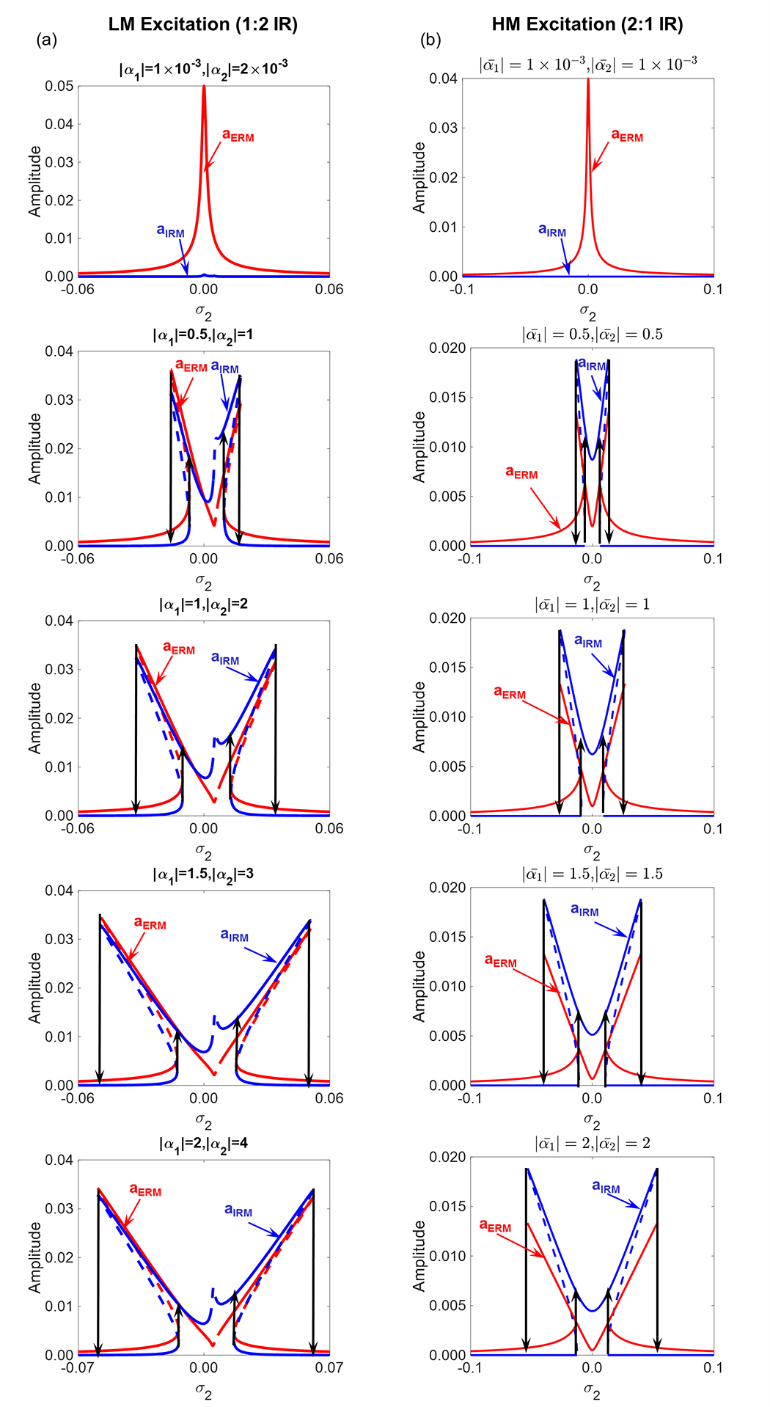


**Figure S5:** (a) Parametric study of the IR amplitude responses in a 1:2 IR system as the nonlinear coefficients increase for the system parameter values of$\omega_{1}=1, \omega_{2}=2, \zeta_{1}=\zeta_{2}=0.001,\lambda=0.1, \sigma_{1}=0.01,w_{F}=5e-4$. (b) Parametric study of the IR amplitude responses in a 2:1 IR system as the nonlinear coefficients increase for the system parameter values of$\omega_{1}=1, \omega_{2}=2, \zeta_{1}=\zeta_{2}=0.001, {\bar{\lambda}=0.2,\sigma}_{1}=0,w_{F}=2e-4$.


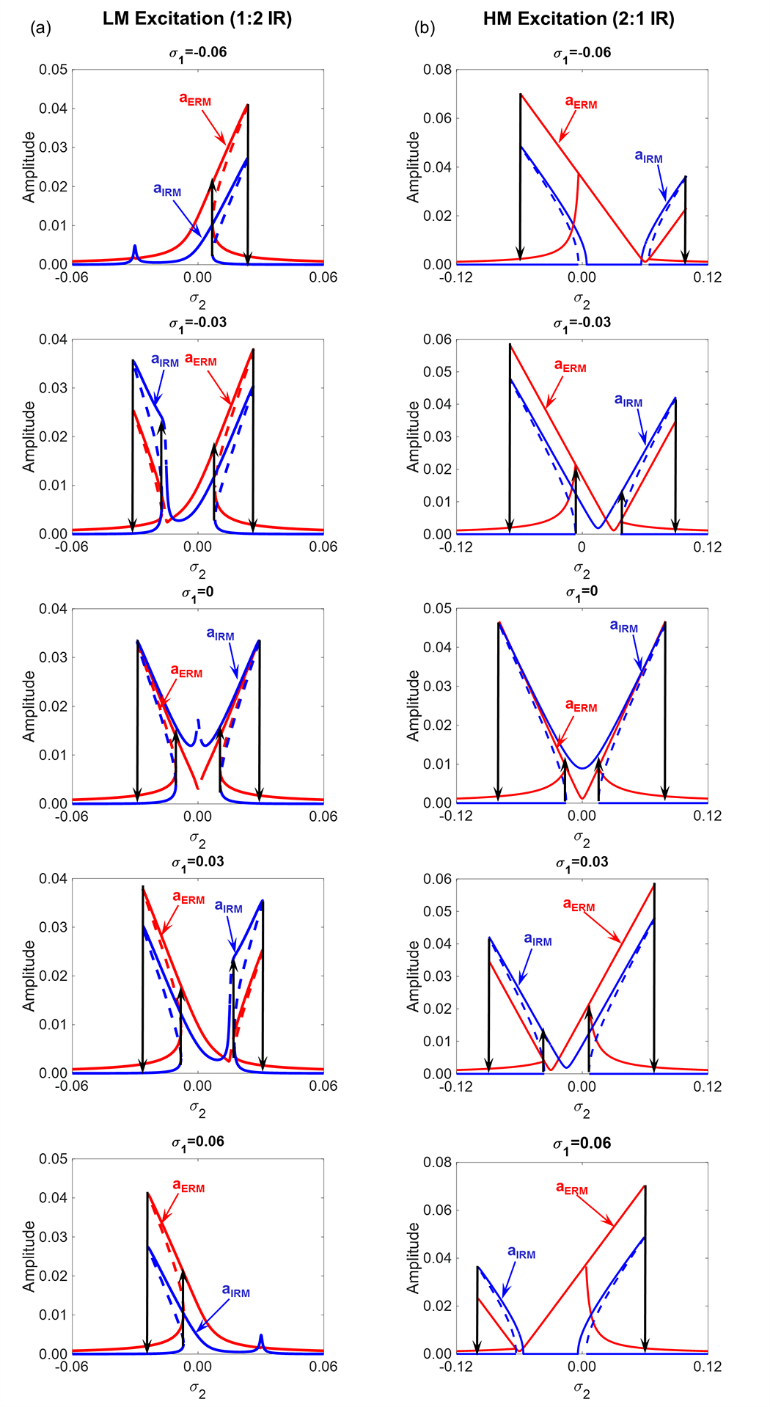


**Figure S6:** (a) Parametric study of the IR amplitude responses in a 1:2 IR system as the internal detuning parameter varies for the system parameter values of$\omega_{1}=1, \omega_{2}=2, \zeta_{1}=\zeta_{2}=0.001, {|\alpha}_{1}|=0.87, {|\alpha}_{2}|=1.75, {\lambda=0.1,w}_{F}=5e-4.$ (b) Parametric study of the IR amplitude responses in a 2:1 IR system as the internal detuning parameter varies for the system parameter values of$\omega_{1}=1, \omega_{2}=2, \zeta_{1}=\zeta_{2}=0.001, \bar{|\alpha}_{1}|=0.85, {|\bar{\alpha}}_{2}|=1.75, \bar{\lambda}=0.2,w_{F}=7e-4$.
